# Cell-specific IL-1R1 regulates the regional heterogeneity of microglial displacement of GABAergic synapses and motor learning ability

**DOI:** 10.1007/s00018-023-05111-0

**Published:** 2024-03-04

**Authors:** Yi You, Da-dao An, Yu-shan Wan, Bai-xiu Zheng, Hai-bin Dai, She-hong Zhang, Xiang-nan Zhang, Rong-rong Wang, Peng Shi, Mingjuan Jin, Yi Wang, Lei Jiang, Zhong Chen, Wei-Wei Hu

**Affiliations:** 1 Department of Pharmacology and Department of Pharmacy of the Second Affiliated Hospital, Key Laboratory of Medical Neurobiology of The Ministry of Health of China, School of Basic Medical Sciences, Zhejiang University School of Medicine, Hangzhou, 310058 China; 2 Key Laboratory of Neuropharmacology and Translational Medicine of Zhejiang Province, Zhejiang Chinese Medical University, Hangzhou, 310053 China; 3 Department of Rehabilitation Medicine, Huzhou Central Hospital, Affiliated Huzhou Hospital, Zhejiang University School of Medicine, Huzhou, 313000 China; 4 Department of Clinical Pharmacy, The First Affiliated Hospital, Zhejiang University School of Medicine, Hangzhou, China; 5 Department of Epidemiology and Biostatistics, Zhejiang University School of Public Health, Hangzhou, 310058 China

**Keywords:** Microglia, Displacement, Synapse, Homeostasis, Motor learning

## Abstract

Microglia regulate synaptic function in various ways, including the microglial displacement of the surrounding GABAergic synapses, which provides important neuroprotection from certain diseases. However, the physiological role and underlying mechanisms of microglial synaptic displacement remain unclear. In this study, we observed that microglia exhibited heterogeneity during the displacement of GABAergic synapses surrounding neuronal soma in different cortical regions under physiological conditions. Through three-dimensional reconstruction, in vitro co-culture, two-photon calcium imaging, and local field potentials recording, we found that IL-1β negatively modulated microglial synaptic displacement to coordinate regional heterogeneity in the motor cortex, which impacted the homeostasis of the neural network and improved motor learning ability. We used the Cre-Loxp system and found that IL-1R1 on glutamatergic neurons, rather than that on microglia or GABAergic neurons, mediated the negative effect of IL-1β on synaptic displacement. This study demonstrates that IL-1β is critical for the regional heterogeneity of synaptic displacement by coordinating different actions of neurons and microglia via IL-1R1, which impacts both neural network homeostasis and motor learning ability. It provides a theoretical basis for elucidating the physiological role and mechanism of microglial displacement of GABAergic synapses.

**Supplementary Information:**

The online version contains supplementary material available at 10.1007/s00018-023-05111-0.

## Introduction

Microglia produce multiple factors and phagocytize debris to participate in physiological or pathological progress [1–3]. In recent years, studies have demonstrated that microglia modulate synaptic function, which is essential for development and homeostasis during adulthood [4, 5]. Microglia are sometimes considered as part of synapses through interacting with neuronal presynaptic and postsynaptic components [6]. Microglial processes engulf synaptic components and reduce synaptic density during the developmental stage, while the impairment of its synaptic pruning leads to abnormal brain function [6–10]. Microglia also modulate the forgetting of remote memories by complement-dependent synapse pruning [11]. Cerebral ischemia prolongs microglia-synapse contacts, which prunes presynaptic boutons [12], and microglia contribute to early synapse loss in Alzheimer's disease through pruning synapses [13, 14]. In addition, microglia can contact the dendrites of neurons to induce actin aggregation and synapse formation as the somatosensory cortex develops [15].

Microglial synaptic displacement was first reported in motor neurons after cutting the facial nerve, i.e., the soma of microglia is closely attached to motor neurons, which displaces synapses around the neuronal soma [16]. In recent years, this microglial behavior in pathological settings has received significant attention. After administering inactivated heat-killed Bacillus Calmette–Guerin bacteria or lipopolysaccharide (LPS), microglia approach neuronal soma to displace surrounding synapses, which can transiently reduce GABAergic inputs and induce anti-apoptotic protein expression [17–19]. We previously demonstrated that during complex febrile seizures, microglial association with neurons reduced GABAergic synaptic transmission by synaptic displacement, but not phagocytosis of synapses, thereby preventing complex febrile seizures [20]. However, whether microglial synaptic displacement contributes to physiological function and its underlying mechanisms are still unclear.

In this study, we observed extent of microglial displacement of GABAergic synapses varied among different cortical regions under physiological states during adulthood. IL-1β can alter synaptic transmission and neuronal excitability [21–23] and induce microglia recruitment [24]. We found that IL-1β/IL-1R1 is also critical for the regional heterogeneity of synaptic displacement, which simultaneously mpacted neural excitability and motor learning ability. We used the Cre-Loxp system and found that IL-1R1 in glutamatergic neurons, rather than that on microglia or GABAergic neurons, mediated the negative effect of IL-1β on synaptic displacement, thereby impacting neural excitability and motor learning.

## Results

### Microglia displayed regional heterogeneity during the displacement of GABAergic synapses surrounding neuronal soma

**Fig. 1:**
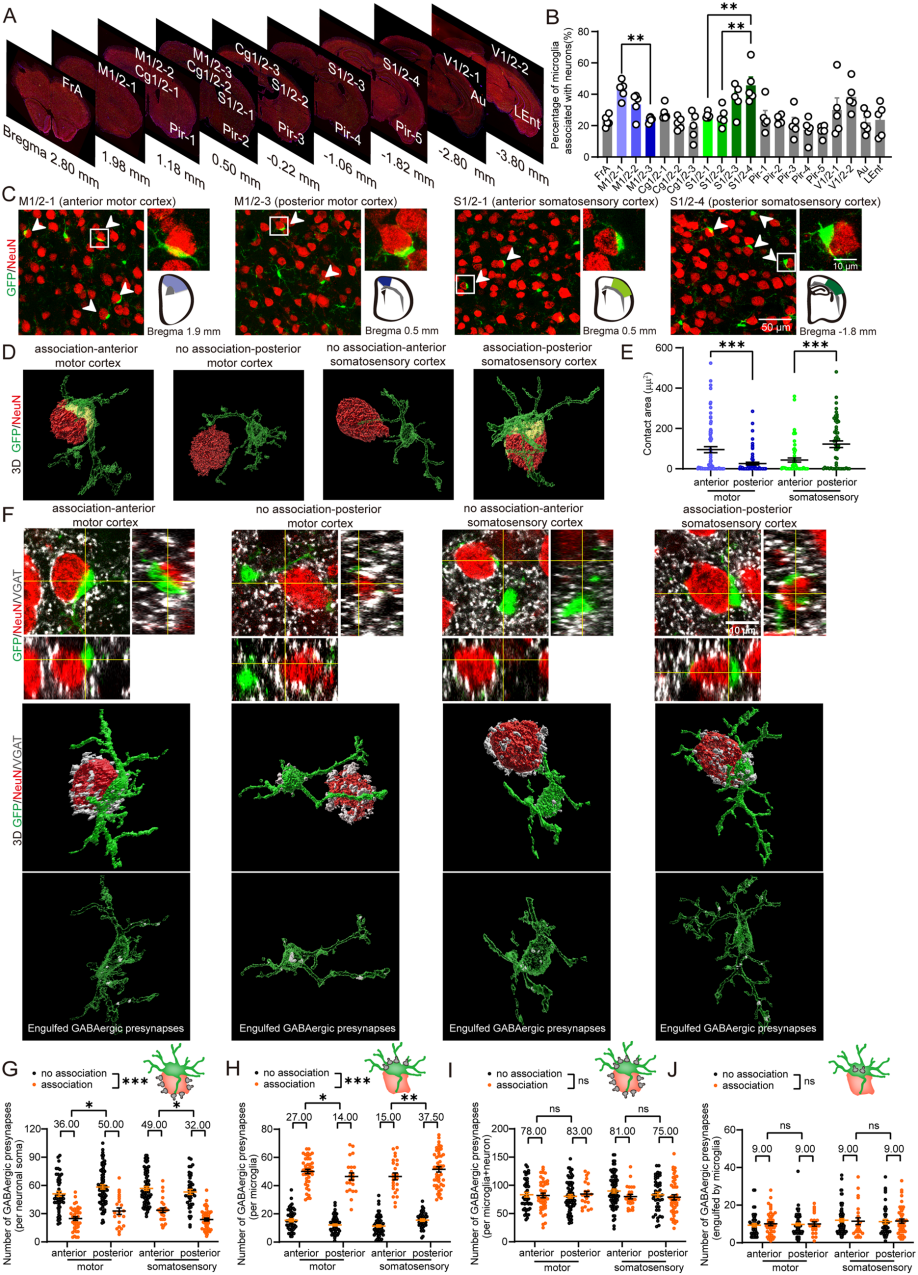
Regional heterogeneity of microglial synaptic displacement in different brain regions. **A** Selected brain regions for observation: anterior association cortex (FrA), the motor cortex (M1/2), cingulate cortex (Cg1/2), piriform cortex (Pir), the somatosensory cortex (S1/2), visual cortex (V1/2), auditory cortex (Au), and lateral entorhinal cortex (Lent). **B** Percentage of microglia extensively associated with neuronal soma in different brain regions. **C** Confocal images of GFP + microglia and NeuN + neuronal soma in *CX3CR1*^*GFP/*+^ mice. The white arrowheads indicate microglia extensively associated with neurons. Cells in the box are shown in enlarged images. Schematic diagrams of the analyzed regions for the motor cortex and somatosensory cortex are shown in the right bottom corners. **D** In 3D reconstructions, yellow areas demarcate the contact area between microglia and neurons, which is quantified in **E**. association-anterior motor cortex: microglia extensively associated with neurons in the anterior motor cortex; no association-posterior motor cortex: microglia not extensively associated with neurons in the posterior motor cortex; no association-anterior somatosensory cortex: microglia not extensively associated with neurons in the anterior somatosensory cortex; association-posterior somatosensory cortex: microglia extensively associated with neurons in posterior somatosensory cortex. **F** Confocal images in orthogonal view (up) and 3D reconstructions (middle and down) of GFP + microglia (green), VGAT + GABAergic synapses (white), and NeuN + neurons (red) in *CX3CR1*^*GFP/*+^ mice. Middle: 3D reconstructions of VGAT + GABAergic synapses around each neuronal and microglial soma. Down: 3D reconstructions of VGAT + GABAergic synapses engulfed in microglia (semitransparent green). **G** The number of GABAergic synapses per neuronal soma that were closest to microglia. The median data are shown above the group. **H** The number of GABAergic synapses around each microglial soma. The median data are shown above the group. **I** The number of GABAergic synapses around each pair of microglial and neuronal soma, which are closest in location. **J** The number of GABAergic synapses engulfed by microglia. Association: extensively association between microglia and neurons; no association: no extensive association between microglia and neurons. *n* = 90–110 cells from 5 mice. One-way ANOVA post hoc Tukey's test was applied for **B**, and Generalized linear mixed model post hoc Bonferroni's test was applied for **E**, **G**–**J**. *P < 0.05, **P < 0.01, ***P < 0.001. The exact description of statistics and groups compared were seen in Table 3

Although microglia dynamically respond to external environment [25], they typically have homogenous cell density and cell morphology in different regions of the cerebral cortex [26, 27]. Recent studies have uncovered differences in the transcriptomes of microglia in different brain regions [28], but little is known about the spatial heterogeneity of microglia. In this study, we utilized adult *CX3CR1*^*GFP/*+^ mice, in which microglia express a green fluorescent protein (GFP), to observe the microglial synaptic displacement in multiple cortical brain regions. First, we calculated the percentage of microglia that were extensively associated with neuronal soma (covering > 25% of the neuronal circumference) [20] in the anterior association cortex (FrA), the motor cortex (M1/2), the cingulate cortex (Cg1/2), the piriform cortex (Pir), the somatosensory cortex (S1/2), the visual cortex (V1/2), the auditory cortex (Au), and the lateral entorhinal cortex (Lent) (Fig. 1a–c). We found that the extensive association between microglia and neuronal soma varied in different cortical regions or even within the same cortical functional region, as indicated by a higher proportion of association at the anterior end of the motor cortex compared to its posterior end and a lower proportion of association at the anterior end of the somatosensory cortex compared to its posterior end (Fig. 1b). There was an equal number of microglia across different cortical regions (Fig. S2A).

Three-dimensional (3D) reconstruction was used to observe the contact between microglia and neurons, and we found that the extensive association between the analyses from 2D confocal images and 3D-reconstruction images were correlated (Fig. 1d, Video S1, Fig. S1A). As extensive association was defined as > 25% microglial coverage of the neuronal circumference in 2D images, a correlated extensive association in 3D-reconstruction images was defined as 13.27% microglial coverage of the neuronal soma (Fig. S1A). Based on these criteria, we also observed a similar phenotype of microglia that extensively associated with neurons compared with that in 2D images (Fig. S1B). Furthermore, the contact area was analyzed in the motor cortex and somatosensory cortex, and we found that the contact area was larger in regions with higher rates of microglial association with neuronal soma (i.e., anterior motor cortex and posterior somatosensory cortex) (Fig. 1d, e). We also checked the surface area of microglia across the motor cortex and somatosensory cortex. The surface area of both microglia and its soma was comparable in the anterior and posterior of the motor cortex or somatosensory cortex (Fig. S1C and S1D), suggesting that the regional heterogeneity in contact area was not induced by the different size of microglia across different brain region. Although microglial processes display a high level of motility [29, 30], we found that the contact area in extensive association was largely contributed by microglial soma but not their processes (Fig. S3B).

Since the surface of neuronal soma was almost covered by GABAergic synapses but not glutamatergic synapses [20, 31], we next observed the GABAergic synapses on neurons, which were closest to microglia, from 3D reconstruction images. GABAergic synapses were distributed on the surface of neuronal soma but were missing where microglia closely interact with neuronal soma (Fig. 1f, Video S2). The quantification of GABAergic synaptic terminals surrounding neuronal soma in four analyzed cortical regions indicated that there were fewer GABAergic synapses for neurons associated with microglia than neurons not associated with microglia (Fig. 1g: no association vs*.* association, *P* < 0.001; Fig. S3C: no association vs*.* association, *P* < 0.001). This suggests that microglial association was related to the reduction of GABAergic synapses around neuronal soma. Meanwhile, we found no difference in the number of GABAergic synapses in neurons associated with microglia in these cortical regions. There were similar numbers of GABAergic synapses for neurons not associated with microglia in these cortical regions (Fig. 1g: comparison among “no association” groups in four regions, *P* = 0.174). However, the number of GABAergic synapses around all neuronal soma (i.e. all analyzed neuron soma closest with microglia), including those associated with microglia or not, was lower in regions with a higher percentage of association (i.e. anterior motor cortex and posterior somatosensory cortex) (Fig. 1g: comparison of the median shown above the four groups, *P* = 0.002). Besides, the density of GABAergic synapses on neuronal soma outside of the contact area was identical among different regions, regardless of whether these neurons were associated with microglia or not (Fig. S3D: comparison of the median shown above the four groups, *P* = 0.187). These results suggest that differences in the percentage of microglial association contribute to differences in the number of GABAergic synapses.

We counted the number of GABAergic synapses surrounding the microglial soma for these groups and found that this association was related to increased number of GABAergic synapses on the microglial surface (Fig. 1h: no association vs*.* association, *P* < 0.001), and that the number of GABAergic synapses on the surface of all microglia was higher in regions with a higher percentage of associations (i.e. anterior motor cortex and posterior somatosensory cortex) (Fig. 1h: comparison of the median shown above the four groups, *P* < 0.001). There was no difference in the number of GABAergic synapses on microglia associated with neurons among these cortical regions, as well as those on microglia not associated with neurons (Fig. 1h: comparison of “no association” groups in four regions, *P* = 0.172; comparison of “association” groups in four regions, *P* = 0.357). We also calculated the number of GABAergic synapses around each pair of microglial and neuronal soma, which are closest in location (Fig. 1i). These numbers were similar regardless of whether they were associated and were comparable among these cortical regions (Fig. 1i: comparison of the median shown above the four groups, *P* = 0.885). Furthermore, the number of GABAergic synapses in microglia was counted to detect the phagocytosis of microglia (Fig. 1j). We observed that microglia displayed similar phagocytosis of GABAergic synapses regardless of whether they were extensively associated with neurons in these cortical regions (Fig. 1j: comparison of the median shown above the four groups, *P* = 0.096; no association vs*.* association, *P* = 0.92). The above data suggest that the different degrees of microglial association and then the displacement of GABAergic synapses, but not phagocytosis, contribute to the different number of GABAergic synapses on neuronal soma in the motor cortex and somatosensory cortex. This displacement could be due to a failure in formation of synapses on neuronal soma after association, or an elimination and translocation of them from neuronal soma to microglial soma. Together, it suggests that there was regional heterogeneity for the displacement of GABAergic synapses on neuronal soma.

### IL-1β/IL-1R1 coordinates the regional heterogeneity of the microglial displacement of GABAergic synapses

**Fig. 2:**
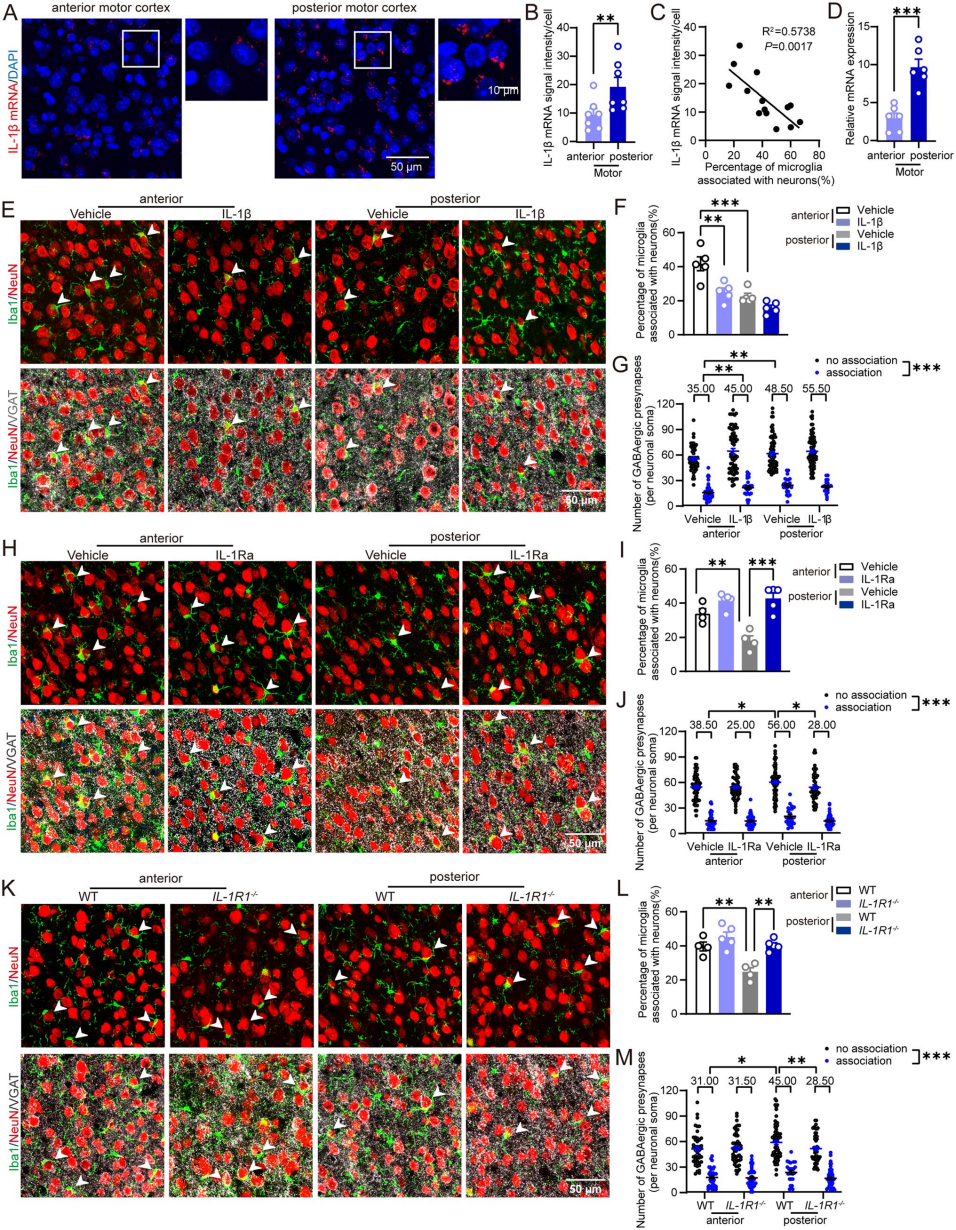
IL-1β/IL-1R1 coordinates the heterogeneity of microglial synapse displacement in the motor cortex. **A** Confocal images of DAPI (blue) and mRNA dots of IL-1β (red) in the anterior motor cortex or posterior motor cortex. **B** RNAscope quantification of IL-1β mRNA expression in the anterior motor cortex and posterior motor cortex. *n* = 7 mice. **C** Correlation analysis between IL-1β mRNA expression and the percentage of microglia extensively associated with neurons. *n* = 14 from 7 mice. **D** Quantification of IL-1β mRNA expression detected by quantitative PCR in the anterior motor cortex and posterior motor cortex. *n* = 6 mice. **E** Confocal images of Iba1 + microglia, VGAT + GABAergic synapses, and NeuN + neuronal soma in IL-1β or vehicle-treated WT mice (injections were made from the lateral ventricle). **F** Percentage of microglia extensively associated with neuronal soma in the IL-1β group and vehicle group. *n* = 5 mice in each group. **G** The numbers of GABAergic synapses around neuronal soma that are associated with microglia or not in IL-1β group and vehicle group. The median data are shown above the group. *n* = 89–99 cells from 5 mice in each group. **H** Confocal images of Iba1 + microglia, VGAT + GABAergic synapses, and NeuN + neurons in IL-1Ra or vehicle-treated WT mice (injections were made from the lateral ventricle). **I** Percentage of microglia extensively associated with neuronal soma in the IL-1Ra group and vehicle group. *n* = 4 mice in each group. **J** The numbers of GABAergic synapses around neuronal soma, which were closest to microglia, in the IL-1Ra group and the vehicle group. Median data are shown above the group. *n* = 93–107 cells from 4 mice in each group. **K** Confocal images of Iba1 + microglia, VGAT + GABAergic synapses, and NenN + neurons in WT mice and *IL-1R1*^*−/−*^ mice. **L** Percentage of microglia extensively associated with neuronal soma in WT mice and *IL-1R1*^*−/−*^ mice. *n* = 4–5 mice in each group. **M** Number of GABAergic synapses around neuronal soma, which were closest to microglia, in WT mice and *IL-1R1*^*−/−*^ mice. Median data are shown above the group. Association: neurons extensively associated with microglia; no association: neurons not extensively associated with microglia. *n* = 87–106 cells from 4–5 mice in each group. Paired *t *test was applied for **B**, **D**, Simple linear regression was applied for **C**, One-way ANOVA post hoc Tukey's test (or Bonferroni's test) was applied for **F**, **I**, **L**, and Generalized linear mixed model *post-hoc* Bonferroni test was applied for **G**, **J**, **M**. *P < 0.05, **P < 0.01, ***P < 0.001. The exact description of statistics and groups compared were seen in Table 3

We investigated the mechanism underlying the regional heterogeneity of microglial displacement of GABAergic synapses. Our previous study found that microglial synaptic displacement induced by LPS was accompanied by the activation of a series of signaling pathways and changes in cytokines, including IL-1Ra (endogenous antagonist of IL-1β) [18]. Moreover, it has been documented that IL-1β can alter synaptic transmission and neuronal excitability [21–23] and induce microglia recruitment [24], so we examined the mRNA expression of IL-1β in the anterior motor cortex and posterior motor cortex using an RNAscope assay and quantitative PCR (Fig. 2a–d). The results demonstrated that the mRNA expression of IL-1β was low in the anterior motor cortex with more synaptic displacement, but high in the posterior motor cortex with less synaptic displacement (Fig. 2b, d). Correlation analysis indicated that IL-1β mRNA expression was negatively correlated with the percentage of extensive association (Fig. 2c), suggesting that IL-1β could be a negative regulator of microglial synaptic displacement.

To verify this hypothesis, we first co-cultured primary microglia labeled by GFP and primary neurons to directly observe the dynamic interaction between microglia and neurons after blocking IL-1β’s functional receptor IL-1R1 by IL-1Ra, an endogenous antagonist of IL-1β [32, 33] (Fig. S4A and S4B, Video S3–S4). We found that most microglia were in an immobile state and only a small group of microglia moved towards or away from neurons (Fig. S4C). After administering IL-1Ra, the percentage of microglia that move towards neuronal soma gradually increased, while the percentage of microglia moving away from neurons and in an immobile state decreased (Fig. S4C). We assigned microglia with three different indexes based on their movement: moving towards neurons (1), staying in an immobile state (0), and moving away from neurons (-1) (Fig. S4D). We found that the association index (the sum of the three indexes) curves were separated after 10 min of IL-1Ra administration and the difference peaked at 20 min, indicating that microglia moved towards neurons at an early phase. We analyzed the microglial distance closest to the neurons and found that IL-1Ra induced a rapid approach of the microglia during early phases, while they were almost immobile in the control stage (Fig. S4E). We then calculated the moving speed of the microglia approaching neurons, when they were located at different distances from the neuronal soma that was classified by different folds of radius (Fig. S4F and S4G). We found that microglial locomotion increased as the distance decreased (Fig. S4G). These results indicate that blocking IL-1R1 promoted microglial associated with neurons, while microglia showed a rapid response with gradually increased speed during this process.

IL-1β was administered into the lateral ventricle of mice through cannula and the percentage of extensive association and the number of GABAergic synapses surrounding neuronal soma closest to microglia were analyzed. The percentage of extensive association and the number of GABAergic synapses surrounding neuronal soma in the vehicle group (Fig. 2f, g) were the same as in the *CX3CR1*^*GFP/*+^ mice (Fig. 1b, g) and wild-type mice (Fig. 2l, m), indicating that this injection from cannula did not change microglial synapse displacement. The results demonstrated that IL-1β significantly decreased the percentage of association (Fig. 2e, f) without affecting the microglial numbers (Fig. S2B) in the anterior motor cortex with an initial higher association percentage. Furthermore, IL-1β did not change the number of GABAergic synapses on neurons associated with microglia as well as those on neurons not associated with microglia, based on 3D reconstruction. To be noted, IL-1β increased the number of GABAergic synapses on all analyzed neuronal soma in the anterior motor cortex (Fig. 2g), along with a decreased proportion of microglial association (Fig. 2f). This suggests that IL-1β stops microglia from approaching the neuronal soma in the anterior motor cortex, which leads to more residual synapses on neuronal soma. In the posterior motor cortex originally with less association, IL-1β had no apparent effect on either the association percentage or the number of GABAergic synapses (Fig. 2f, g). To be noted, the regional heterogeneity of microglial synaptic displacement in the anterior and posterior motor cortex was lost after IL-1β treatment (Fig. 2g). No inflammatory activation of the anterior motor cortex was elicited after IL-1β treatment, as there was no change in the expression of inflammatory signal pathways p-IκBα and p-p38 (Fig. S5).

We then blocked IL-1R1 by administering IL-1Ra into the lateral ventricle of mice through cannula in vivo and using IL-1R1 knockout mice. The results demonstrated that IL-1Ra induced a significant increase in the percentage of association and a reduction in the number of synapses on neurons closest to microglia in the posterior motor cortex, but not in the anterior motor cortex. Both the microglial association and the number of synapses on neurons reached a comparable level in the anterior and posterior motor cortex after IL-1Ra administration (Fig. 2h–j, Fig. S6), indicating the loss of regional heterogeneity of microglial synaptic displacement. Similarly, in IL-1R1 knockout mice, the percentage of association increased while the number of synapses on neurons decreased in the posterior motor cortex, both of which reached levels comparable to those in the anterior motor cortex (Fig. 2k–m). Besides, the number of microglia were identical among groups, as were the number of GABAergic synapses for neurons associated with microglia or not (Fig. S2C and S2D, Fig. 2j, m). These results suggest that IL-1β is a negative factor modulating microglial synaptic displacement via IL-1R1, which is also responsible for its heterogeneity in the motor cortex.

### IL-1β/IL-1R1 modulates neural network homeostasis and motor learning ability by regulating microglial synaptic displacement

**Fig. 3:**
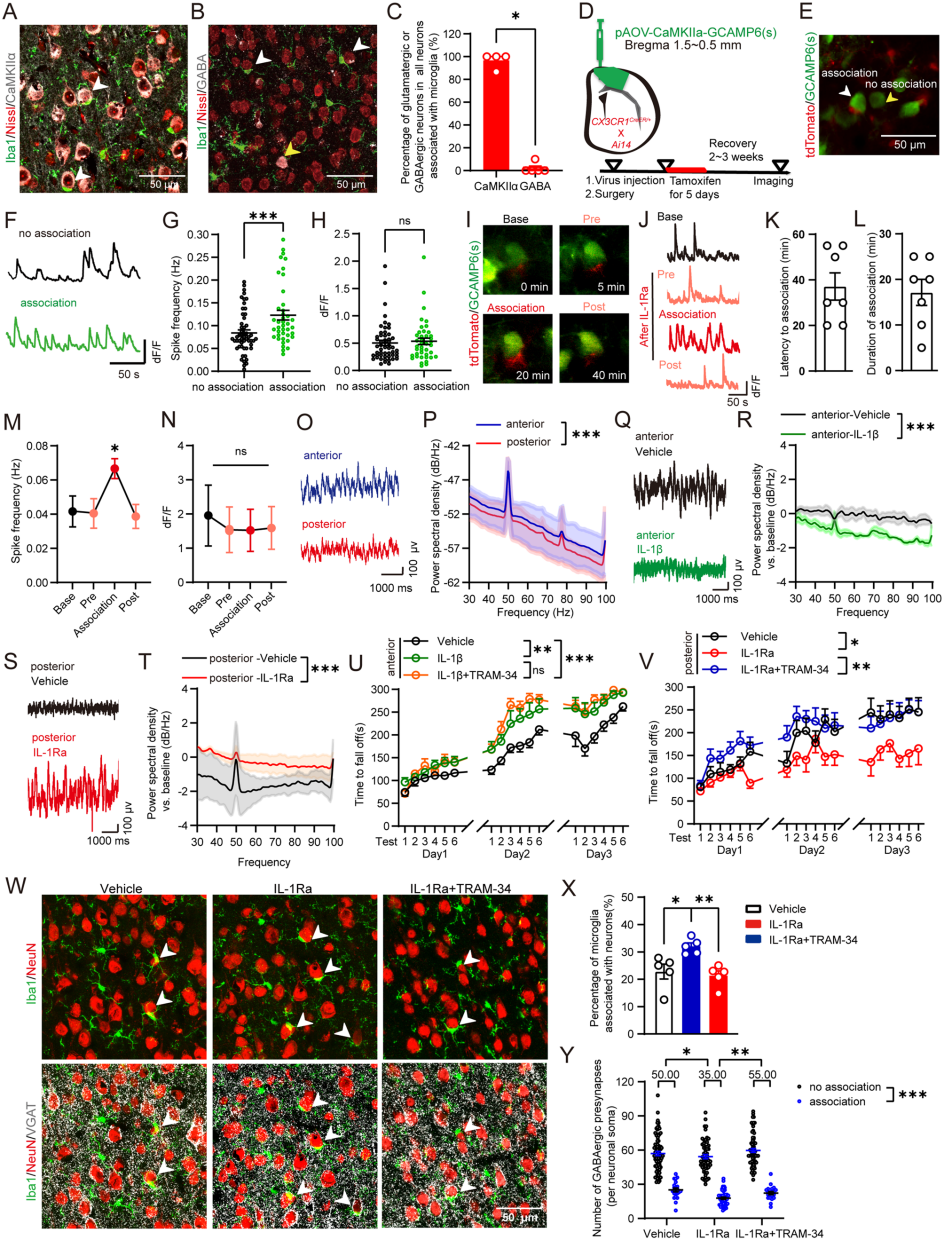
Microglial synaptic displacement maintains homeostasis of neural network and motor learning ability modulated by IL-1β/IL-1R1. **A** Confocal images of Iba1 + microglia, Nissl + neurons, and CaMKIIα + glutamatergic neurons in the posterior motor cortex of C57 mice. White arrowheads indicate glutamatergic neurons extensively associated with microglia. **B** Confocal images of Iba1 + microglia, Nissl + neurons, and GABA + neurons in the posterior motor cortex. The white arrowheads indicate the neurons extensively associated with microglia, while the yellow arrowheads indicate the GABA + neurons not extensively associated with microglia. **C** The percentage of microglia extensively associated with CaMKIIα + glutamatergic or GABAergic neurons in the posterior motor cortex. *n* = 4–5 mice in each group. **D** Protocol for surgery and in vivo two-photon imaging. After pAOV-CaMKIIa-GCAMP6(s) virus injection and two-photon surgery, *CX3CR1*^*CreER/*+^*;Ai14* mice were administrated with tamoxifen (2 mg/day) for 5 consecutive days, and calcium imaging was performed after the mice recovered for another 2–3 weeks. **E** Image of GCAMP6(s) labeled glutamatergic neurons (green) either associated with microglia (red) or not. The white arrowhead indicates neurons extensively associated with microglia and the yellow arrowhead indicates neurons not extensively associated with microglia. **F** Calcium traces of neurons which are associated with microglia or not. **G** Frequency of calcium transients of glutamatergic neurons associated with microglia or not. *n* = 41–53 cells from 5 mice. **H** Change of calcium intensity of glutamatergic neurons which is associated with microglia or not. **I** Image of GCAMP6(s) labeled glutamatergic neurons (green) and microglia (red) gradually extensively associated with the neuronal soma over time after administration of IL-1Ra from the lateral ventricle. **J** Calcium traces of neurons are gradually extensively associated with microglia over time after administration of IL-1Ra. **K** Latency of microglia is extensively associated with neuronal soma. **L** Duration of microglia extensively associated with neuronal soma. **M** Calcium spikes the frequency of glutamatergic neurons when microglia are gradually extensively associated with the neuronal soma. **N** Change of calcium intensity of glutamatergic neurons when microglia gradually extensively associated with the neuronal soma. *n* = 7 cells from 3 mice. **O** Representative traces of LFP in the anterior motor cortex (anterior) or posterior motor cortex (posterior) in WT mice. **P** The LFP gamma power spectral density in the anterior motor cortex (anterior) or posterior motor cortex (posterior). *n* = 3 mice for each group. **Q** Representative traces of LFP in the anterior motor cortex (anterior) of the vehicle group or IL-1β group in WT mice. **R** The LFP gamma power spectral density in the anterior motor cortex of the vehicle group or IL-1β group (normalized by baseline). *n* = 5 mice for each group. **S** Representative traces of LFP in the posterior motor cortex (posterior) of the vehicle group or IL-1Ra group in WT mice. **T** The LFP gamma power spectral density in the posterior motor cortex after IL-1Ra or vehicle administration (normalized by baseline). *n* = 6 mice for each group. **U** The latency fall off when a vehicle, IL-1β or IL-1β combined with TRAM-34 (i.p.) was administered in the anterior motor cortex of WT mice. *n* = 10–11 mice for each group. **V** The latency to fall off in mice administrated with the vehicle, IL-1Ra, or IL-1Ra in the posterior motor cortex combined with TRAM-34 (i.p.) of WT mice. *n* = 9 mice for each group. **W** Confocal images of Iba1 + microglia, VGAT + GABAergic synapses, and NeuN + neuronal soma in the posterior motor cortex of the vehicle, IL-1Ra, or IL-1Ra group combined with TRAM-34 group in WT mice. White arrowheads indicate microglia extensively associated with neuronal soma. **X** The percentage of microglia extensively associated with neuronal soma in the vehicle, IL-1Ra, or IL-1Ra combined with TRAM-34 group. *n* = 5 mice for each group. **Y** Numbers of GABAergic synapses around neuronal soma, which were closest to microglia, in the vehicle, IL-1Ra, or IL-1Ra group combined with TRAM-34 group. Median data are shown above the group. Association: neurons extensively associated with microglia; no association: neurons not extensively associated with microglia. *n* = 94–106 cells from 5 mice in each group. Mann–Whitney test was applied for **C**, generalized linear mixed model was applied for **G**, **H**, **Y**, Friedman test post hoc Dunn's test was applied for **M**, **N**, Two-way ANOVA test was applied for **P**, **R**, **T**, Repeated measures two-way ANOVA test was applied for **U**, **V**, and One-way ANOVA post hoc Tukey's test was applied for **X**. *P < 0.05, **P < 0.01, ***P < 0.001. The exact description of statistics and groups compared were seen in Table 3

To examine the physiological role of microglial synaptic displacement, we first labeled cortical excitatory glutamatergic neurons or inhibitory neurons with CaMKIIα (Fig. 3a) or GABA (Fig. 3b), respectively, to identify which neurons the microglia associated with. The results demonstrated that microglia were primarily extensively associated with glutamatergic neurons in the posterior motor cortex, while few GABAergic neurons were associated with microglia (Fig. 3c). This indicates that microglia primarily approach glutamatergic neurons under adult physiological conditions. We then studied the effect of microglial synaptic displacement on individual neuronal homeostasis using two-photon imaging, which allows the calcium imaging of neuronal activity in mice, assisted by a green fluorescent calcium indicator and high-frequency resonance scanning. The pAOV-CaMKIIα-GcAMP6(s) virus was injected into the posterior motor cortex of *CX3CR1*^*CreER/*+^*; Ai14* mice expressing red fluorescent in microglia to observe the activity of glutamatergic neurons associated with microglia (Fig. 3d–h, Video S5). We found that neurons extensively associated with microglia displayed a higher frequency of calcium spikes than neurons that were not associated with microglia (Fig. 3g), but found no difference in the amplitude of calcium spikes (Fig. 3h). After administration of IL-1Ra, the average latency for microglia to extensively associate with neuronal soma was about 37 min, while the duration of extensive association was about 17 min (Fig. 3i, k, l). Importantly, the calcium spikes frequency of neurons was increased after microglia extensively attaching neuronal soma but recovered when microglial association was attenuated (Fig. 3j, m, n, Video S6). It suggests that microglia displace GABAergic synapses around neuronal soma to enhance neuronal excitability, which could be negatively regulated by IL-1R1.

To study whether regional heterogeneity during the displacement of GABAergic synapses can lead to differences in the excitability of neural networks in different cortical regions, we recorded local field potentials (LFP) at both the anterior motor cortex and the posterior motor cortex. The LFP gamma power spectral density of free-moving mice were analyzed since it is generally considered to be associated with GABAergic transmission [34–36], and the gamma oscillations are altered by the synaptic connectivity between excitatory and inhibitory neurons [35]. We observed higher energy in the anterior motor cortex compared with the posterior motor cortex (Fig. 3o, p), which is identical to the degree of microglial synaptic displacement. These results suggest that the synaptic displacement of microglia affects the excitability of individual neurons and the neural network. Importantly, the regional heterogeneity of the displacement of GABAergic synapses may result in various levels of homeostasis of neural networks in different brain regions.

To investigate the effects of IL-1β/IL-1Ra on neural network homeostasis after their modulation of microglial synaptic displacement, we recorded the LFP at the anterior or posterior motor cortex after administering IL-1β (i.c.v.) or IL-1Ra (i.c.v.), respectively. We found that IL-1β reduced the LFP gamma power spectral density in the anterior motor cortex (normalized by baseline, Fig. 3q, r), where IL-1β also reduced microglial displacement (Fig. 2f, g). In contrast, IL-1Ra markedly increased the gamma power spectral density in the posterior motor cortex (Fig. 3s, t), where IL-1Ra significantly increased microglial displacement (Fig. 2i, j). These results suggest that IL-1/IL-1R1 is engaged in maintaining the neural network homeostasis following regulation of microglial synaptic displacement.

While microglia-mediated synaptic displacement prevents febrile seizures and can alleviate the effects of brain injuries [18, 20], their physiological role is unknown. We found that administering IL-1β or IL-1Ra at the anterior or posterior motor cortex, respectively, did not change the locomotor activity of mice, including total distance, motion speed, and slow or fast motion time either in an open field (Fig. S7A, S7B, S7J, S7K) or in a home cage (Fig. S7C–S7H, S7L–S7Q). This suggests that their motor execution ability may not be modulated by IL-1β/IL-1R1. Previous studies suggested that the motor cortex also plays a critical role in motor learning [37, 38], which is often closely related to synaptic plasticity and neuronal activity [37–39]. We then employed the accelerating rotarod model to detect motor learning ability after locally administering IL-1β or IL-1Ra in the anterior motor cortex or posterior motor cortex. We found that locally injecting IL-1β in the anterior motor cortex significantly enhanced motor learning ability, as indicated by upregulation of latency falling off the rotarod during consecutive 3-day tests, while IL-1β had no effect when injected in the posterior motor cortex (Fig. 3u, Fig. S7I). In contrast, locally injecting IL-1Ra in the posterior motor cortex significantly impaired motor learning ability, as indicated by the downregulation of the latent curve compared with the vehicle group, while IL-1Ra had no effect when injected in the anterior motor cortex (Fig. 3v, Fig. S7R). To further verify whether IL-1β/IL-1Ra affects motor learning ability in mice due to microglial synaptic displacement, we used TRAM-34, which can inhibit microglial synaptic displacement [20]. We found that TRAM-34 decreased the percentage of microglial association but increased the number of GABAergic synapses around neuronal soma, indicating that it reversed the increase of microglial displacement induced by IL-1Ra (Fig. 3x, y). TRAM-34 also completely reversed the impairment in motor learning ability caused by IL-1Ra (Fig. 3v). Together, the above data suggest that IL-1β/IL-1R1 governs microglial synaptic displacement to distinctively modulate neural network excitability and motor learning ability in different regions of the motor cortex.

### IL-1R1 on glutamatergic neurons, rather than that on microglia or GABAergic neurons, mediated the negative effect of IL-1β on synaptic displacement

**Fig. 4:**
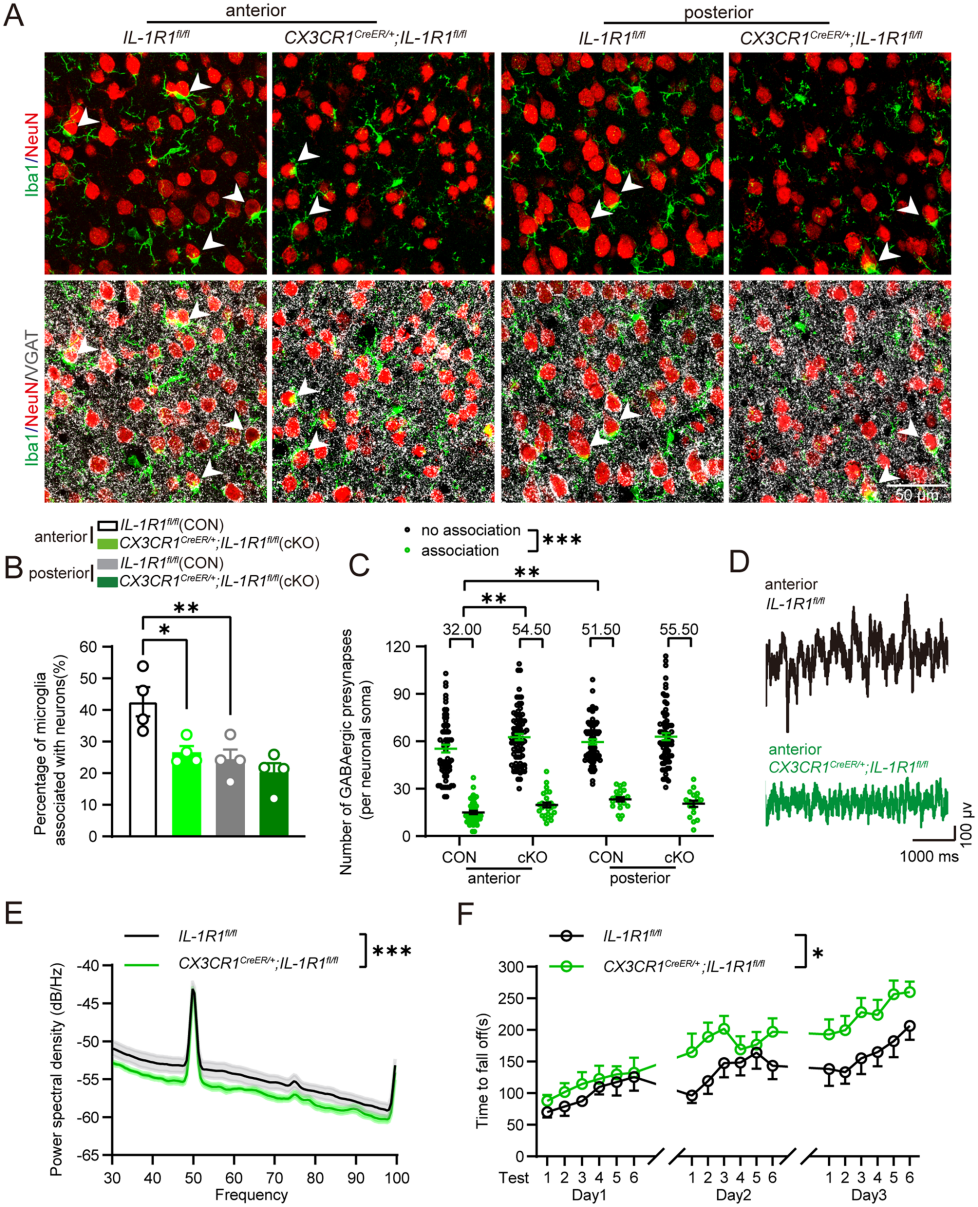
Selective deficit of IL-1R1 in microglia decreases microglial synaptic displacement and improves motor learning. **A** Confocal images of Iba1 + microglia, NeuN + neurons and VGAT + GABAergic synapses in the anterior motor cortex (anterior) and posterior motor cortex (posterior) of control mice (*IL-1R1*^*fl/fl*^) and cKO mice (*CX3CR1*^*CreER/*+^*; IL-1R1*^*fl/fl*^). White arrowheads indicate microglia extensively associated with neuronal soma. **B** Percentage of microglia extensively associated with neuronal soma in control mice (*IL-1R1*^*fl/fl*^) and cKO mice (*CX3CR1*^*CreER/*+^*; IL-1R1*^*fl/fl*^). *n* = 4 mice for each group. **C** Numbers of GABAergic synapses around neuronal soma, which were closest to microglia, in control mice (*IL-1R1*^*fl/fl*^, CON) and *CX3CR1*^*CreER/*+^*; IL-1R1*^*fl/fl*^ mice (cKO) under 3D reconstruction. Median data are shown above the group. *n* = 90–107 cells from 4 mice in each group. Association: neurons extensively associated with microglia; no association: neurons not extensively associated with microglia. **D** Representative LFP trace in anterior motor cortex of control mice (*IL-1R1*^*fl/fl*^) and cKO mice (*CX3CR1*^*CreER/*+^*; IL-1R1*^*fl/fl*^). **E** The LFP gamma power spectral density in control mice (*IL-1R1*^*fl/fl*^) and cKO mice (*CX3CR1*^*CreER/*+^*; IL-1R1*^*fl/fl*^). *n* = 5–6 mice for each group. **F** The latency to fall off in rotarod tests in control mice (*IL-1R1*^*fl/fl*^) and cKO mice (*CX3CR1*^*CreER/*+^*; IL-1R1*^*fl/fl*^). *n* = 12 mice for each group. One-way ANOVA post hoc Tukey's test was applied for **B**, Generalized linear mixed model post hoc Bonferroni's test was applied for **C**, Two-way ANOVA test was applied for **E**, and Repeated measures two-way ANOVA test was applied for **F**. *P < 0.05, **P < 0.01, ***P < 0.001. The exact description of statistics and groups compared were seen in Table 3

Although neuron expresses higher level of IL-1R1 compared with microglia, IL-1R1 on both cells were involved in the neuroinflammation and neurodegenerative diseases [40–45]. To clarify the role of IL-1R1 in microglia or glutamatergic neurons in synaptic displacement, we crossed *IL-1R1*^*fl/fl*^ mice to *CX3CR1*^*CreER/*+^ mice and *CaMKIIα*^*Cre*^ mice to specific deficit *IL-1R1* expression in microglia and glutamatergic neurons, respectively (Figure S8A–E). Interestingly, we found that IL-1R1 deficits in microglia resulted in fewer microglia close to the neuronal soma (Fig. 4a, b) and an increase in the number of GABAergic synapses around neuronal soma closest to microglia in the anterior motor cortex, but did not affect the posterior motor cortex (Fig. 4c). Analysis of the LFP gamma power spectral density revealed that IL-1R1 deficits in microglia decreased overall neural network excitability in the anterior motor cortex (Fig. 4d, e). Moreover, motor learning ability was significantly enhanced in *CX3CR1*^*CreER/*+^*;IL-1R1*^*fl/fl*^ mice compared with the control (Fig. 4f). This suggests that IL-1R1 deficits on microglia induce an opposite effect compared to entire IL-1R1 deficits or application of the antagonist IL-1Ra concerning microglial synaptic displacement, neural network excitability, and motor learning ability (Figs. 2h–m, 3s–v, 4).

**Fig. 5:**
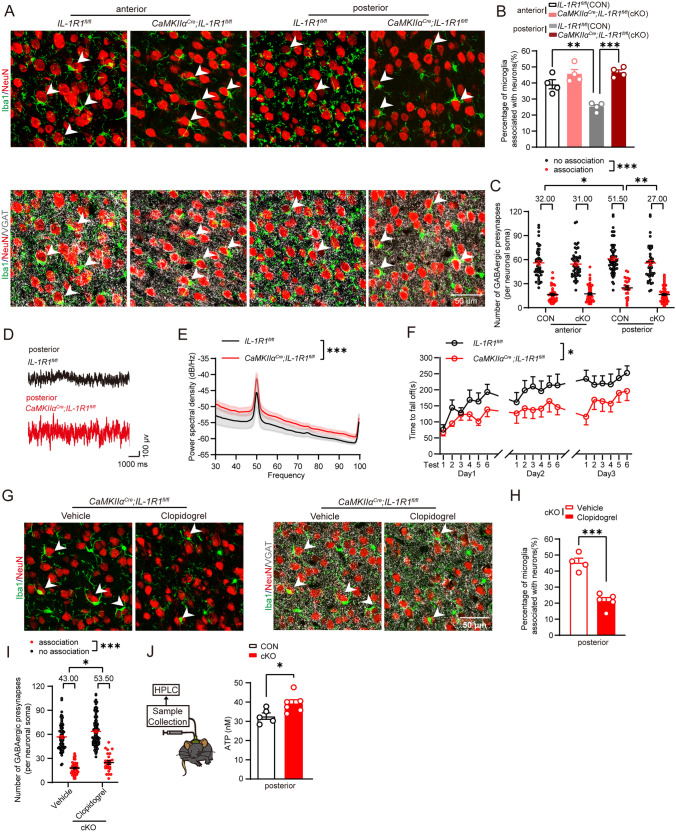
Selective deficit of IL-1R1 in glutamatergic neurons leads to increased synaptic displacement and impairs motor learning. **A** Confocal images of Iba1 + microglia, NeuN + neurons and VGAT + GABAergic synapses in the anterior motor cortex (anterior) and posterior motor cortex (posterior) of control mice *(IL-1R1*^*fl/fl*^) and *CaMKIIα*^*Cre*^*;IL-1R1*^*fl/fl*^ mice. White arrowheads indicate microglia extensively associated with neuronal soma. **B** Percentage of microglia extensively associated with a neuron in control mice (*IL-1R1*^*fl/fl*^) and *CaMKIIα*^*Cre*^*;IL-1R1*^*fl/fl*^ mice. *n* = 4 mice for each group. **C** Numbers of GABAergic synapses around neuronal soma, which were closest to microglia, in control mice (*IL-1R1*^*fl/fl*^, CON) and *CaMKIIα*^*Cre*^*;IL-1R1*^*fl/fl*^ mice (cKO) under 3D reconstruction. Median data are shown above the group. *n* = 94–109 cells from 4 mice in each group. **D** Representative LFP traces in the posterior motor cortex of control mice (*IL-1R1*^*fl/fl*^) and *CaMKIIα*^*Cre*^*;IL-1R1*^*fl/fl*^ mice. **E** The LFP gamma power spectral density in control mice *(IL-1R1*^*fl/fl*^) and *CaMKIIα*^*Cre*^*;IL-1R1*^*fl/fl*^ mice (30–100 Hz). *n* = 5 mice for each group. **F** The latency to fall off in rotarod tests in control mice (*IL-1R1*^*fl/fl*^) and *CaMKIIα*^*Cre*^*;IL-1R1*^*fl/fl*^ mice. *n* = 9–10 mice for each group. **G** Confocal images of Iba1 + microglia, NeuN + neurons and VGAT + GABAergic synapses in posterior motor cortex in vehicle or clopidogrel treated *CaMKIIα*^*Cre*^*;IL-1R1*^*fl/fl*^ mice. **H** Percentage of microglia extensively associated with neuronal soma in vehicle or clopidogrel treated *CaMKIIα*^*Cre*^*;IL-1R1*^*fl/fl*^ mice. *n* = 4–5 mice for each group. **I** Numbers of GABAergic synapses around neuronal soma, which were closest to microglia, in vehicle or clopidogrel treated *CaMKIIα*^*Cre*^*;IL-1R1*^*fl/fl*^ mice. Median data are shown above the group. *n* = 128–130 cells from 4–5 mice for each group. **J** Quantitative analysis of ATP with microdialysis of control mice (*IL-1R1*^*fl/fl*^) and *CaMKIIα*^*Cre*^*;IL-1R1*^*fl/fl*^ mice in the posterior motor cortex. *n* = 5–6 in each group. Association: neurons extensively associated with microglia; no association: neurons not extensively associated with microglia. One-way ANOVA post hoc Tukey's test was applied for **B**, Generalized linear mixed model post hoc Bonferroni's test was applied for **C**, **I**, Two-way ANOVA test was applied for **E**, Repeated measures two-way ANOVA test was applied for **F**, and unpaired *t* test was applied for **H**, **J**. *P < 0.05, ***P < 0.001. The exact description of statistics and groups compared were seen in Table 3

In contrast, IL-1R1 deficits in glutamatergic neurons resulted in significantly increased extensive microglial associations (Fig. 5a, b) and a decreased number of GABAergic synapses around neuronal soma closest to microglia only in the posterior motor cortex (Fig. 5c and Fig. S9). This suggests that IL-1R1 deficits in glutamatergic neurons promote microglia to associate with and displace synapses around neuronal soma, which is consistent with the general deficit of IL-1R1 or application of the antagonist IL-1Ra (Fig. 2h–m). Interestingly, differences in extensive associations and the number of GABAergic synapses in the anterior motor cortex and posterior motor cortex were not observed in *CaMKIIα*^*Cre*^*;IL-1R1*^*fl/fl*^ mice (Fig. 5c). *CaMKIIα*^*Cre*^*;IL-1R1*^*fl/fl*^ mice also displayed increased gamma power spectral density in LFP recordings of the posterior motor cortex region (Fig. 5d, e), but impaired motor learning ability compared with the control (Fig. 5f), which was comparable to that in *IL-1R1*^*−/−*^ mice. Since GABAergic synapses surrounding neuronal soma were displaced by microglia, *VGAT*^*Cre*^*;IL-1R1*^*fl/fl*^ mice were generated to selectively induce an IL-1R1 deficit in GABAergic neurons (Fig. S8F–S8G). We found that the percentage of microglia associated with neurons was not altered either in the anterior motor cortex or the posterior motor cortex (Fig. S10B), suggesting that IL-1R1 in GABAergic neurons may not contribute to microglia-mediated synaptic displacement. Additionally, the number of microglia was unchanged in *CX3CR1*^*CreER/*+^*;IL-1R1*^*fl/fl*^, *CaMKIIα*^*Cre*^*;IL-1R1*^*fl/fl*^, and *VGAT*^*Cre*^*;IL-1R1*^*fl/fl*^ mice (Fig. S2F and S2G, S10C). This suggests that IL-1R1 in microglia and neurons play opposite roles in the microglia-mediated synaptic displacement. To be noted, IL-1R1 on glutamatergic neurons, rather than that on microglia or GABAergic neurons, mediated the negative effect of IL-1β on synaptic displacement, thereby impacting neural excitability and motor learning.

It has been reported that microglia are attracted by ATP sources [46, 47] and that neurons can release ATP from the vesicular or nonvesicular pathway, such as axons and other nonsynaptic regions [48, 49]. Our previous studies found that in complex febrile seizures, P2Y_12_ receptors on microglia responding to ATP promote microglia to displace GABAergic synapses [20], leading us to question whether IL-1R1 on glutamatergic neurons affects ATP synthesis or release to modulate microglial displacement. We injected clopidogrel, a P2Y_12_ receptor antagonist, into *CaMKIIα*^*Cre*^*;IL-1R1*^*fl/fl*^ mice and found that it abrogated increases in microglial association and decreases in the number of GABAergic synapses around all neuronal soma in the posterior motor cortex (Fig. 5g–i). Increases in extracellular ATP level were observed in the posterior motor cortex of *CaMKIIα*^*Cre*^*;IL-1R1*^*fl/fl*^ mice through microdialysis (Fig. 5j). This suggests that IL-1R1 in glutamatergic neurons suppresses ATP production, which impedes microglial association with neuronal soma and the displacement of GABAergic synapses via P2Y_12_ receptors.

## Discussion

At various physiological stages, microglia’s pruning of synapses by phagocytosis has received much attention [6–9, 11]. Previous studies of microglia-mediated synaptic displacement have focused on pathological conditions such as nerve injuries, LPS-induced neuroinflammation, and febrile seizures [17, 18, 20]. However, its physiological role and regulatory mechanism remain unclear. This study reveals that microglial displacement of GABAergic synapses displays regional heterogeneity, and maintains homeostasis in the neural network and motor learning ability in physiological states. We identified a critical regulatory mechanism of microglial synaptic displacement: IL-1β, mainly released by neurons, acts on neuronal IL-1R1 to negatively modulate microglial synaptic displacement with regional heterogeneity in the motor cortex. This study provides a new paradigm from which microglia can regulate synaptic plasticity and neural network homeostasis.

### Microglial displacement of GABAergic synapses is regionally heterogeneous to modulate motor learning ability

Previous studies have observed some microglia close to the neuronal soma as satellite microglia, whose number could alter after pathological insults [17, 18, 50, 51], indicating that the microglial association is not static, but dynamic in response to the microenvironment. However, the regulation mechanism and function of satellite microglia remains unclear. Our results may explain the regulation and function of satellite microglia by displacing GABAergic synapses. We found that the extent of microglial displacement of GABAergic synapses varied among different cortical regions under physiological states in adulthood, though the number of microglia was identical. There is more microglial displacement in the anterior motor cortex and the posterior somatosensory cortex, but less in the posterior motor cortex and the anterior somatosensory cortex. This difference in microglial displacement is due to different amounts of microglia associating with neuronal soma and, based on the following evidence: (1) the neuron associated with microglia had less GABAergic synapses and the number of GABAergic synapses on all neuronal soma, regardless of whether they are associated with microglia or not, were negatively correlated with the different association percentage in motor and somatosensory cortexes. (2) change of microglial association by regulating IL-1R1 also altered the amounts of GABAergic synapses around all neuronal soma. (3) the density of GABAergic synapses on neuronal soma outside of the contact area was identical, regardless of whether these neurons were associated with microglia or not. (4) more GABAergic synapses were observed on microglial soma, along with less GABAergic synapses on neuronal soma in associated groups. The total number of GABAergic synapses on a close pair of microglial and neuronal soma were identical in both the associated groups and the not associated groups. (5) the phagocytosis of synapses is not responsible for the difference in the number of GABAergic synapses on all neuronal soma. These results suggest that microglia associate with neuronal soma to displace surrounding GABAergic synapses, rather than attaching neurons with less GABAergic synapses. Importantly, the two-photon imaging indicated that microglial association increased neuronal activity, supporting the second hypothesis that microglial association confers the translocation of GABAergic synapses to result in synaptic displacement.

Microglial heterogeneity can enable localized neural homeostasis relying on brain regions. We found that the difference in microglial displacement of GABAergic synapses may result in distinctive activity of local neural networks in the anterior and posterior motor cortex. While the primary action of the motor cortex is motor execution [52, 53], the motor cortex also plays a role in motor learning [37, 39]. During motor learning, the excitability of neurons was either increased or decreased in different areas of the motor cortex, and the formation of spines and long-term enhancement was also observed [54, 55]. It has been reported that either optogenetic enhancement or inhibition of a subtype of GABAergic neurons in the motor cortex damaged the learning ability through destabilizing or hyperstabilizing spines [56]. So, moderate GABAergic transmission in motor cortex may be indispensable to spine plasticity and motor learning. Our study suggests that microglial displacement affects GABAergic transmission and neural network excitability, which may impact spine plasticity and motor learning. Furthermore, we found that IL-1β/IL-1R1 can govern the regional heterogeneity of microglial displacement and the excitatory-inhibitory balance of neural networks in the anterior and posterior motor cortex to fine-tune motor learning, but not the motor execution. This study provides insights into the physiological modulation of motor learning by microglial synaptic displacement, in addition to spine plasticity.

### IL-1β/IL-1R1 are negative signals for coordinating regional heterogeneity of microglial displacement of GABAergic synapses

The mechanism of microglia-mediated synaptic displacement is not well defined. After LPS administration, microglia were found to undergo synaptic displacement accompanied by alterations in a range of molecules, including Ym1 (also known as chi3l3), SOCS3, IL-4Ra, PTPRC, CD163, IL-1Ra, Mrc1, and Arg1 [17], but it is unknown whether these molecules are involved in the regulation of microglial synaptic displacement. We previously found that the activation of P2Y_12_ receptors in complex febrile seizures promoted displacement of GABAergic synapses and inhibited febrile seizures [20], but the negative factors that affect microglial synaptic displacement remain unclear. IL-1β is ubiquitously expressed in various cells, but previous studies have often focused on its role in neuroinflammatory response. IL-1β increased neuron excitability [57–59], which may contribute to seizures [60–62], multiple sclerosis [63], and ischemia [64]. However, some studies found that IL-1β inhibited long-term potentiation in the hippocampus in inflammatory responses elicited by LPS or hepatitis B vaccination [65, 66], suggesting complicated effects of IL-1β on neural excitability under different pathological conditions.

Our study reveals a regulatory role for IL-1β in synaptic plasticity and neural excitability under physiological conditions: IL-1β alters synaptic transmission by negatively modifying microglial synaptic displacement via IL-1R1 based on both in vitro and in vivo experiments (Figs. 2, 3). Interestingly, IL-1β/IL-1R1 modulated microglial synaptic displacement within a certain range under physiological conditions. Administering IL-1β decreased synaptic displacement in the anterior motor cortex with initial low expression of IL-1β, while IL-1β had no significant effect on the posterior motor cortex where the expression of IL-1β was high. In contrast, IL-1Ra or IL-1R1 knockout only increased synaptic displacement in the posterior motor cortex without affecting the anterior motor cortex. One possible reason is that only a subgroup of microglia or neurons can be modulated by IL-1β or IL-1Ra during synaptic displacement. Alternatively, other factors could be working with IL-1β to confine the range of microglial displacement. Additionally, IL-1β is also confined at a limited range to modulate motor learning ability, since IL-1β only improves motor learning ability in the anterior motor cortex but not the posterior motor cortex, while IL-1Ra only impedes motor learning ability in the posterior motor cortex but not anterior motor cortex. These limited effects of IL-1β may differ from its role in neuroinflammation with a dramatic increase of IL-1β expression. Nevertheless, our results indicate that differences in IL-1β levels in different cortical regions could be responsible for the regional heterogeneity of microglial displacement, generating distinctive neural excitability that can affect neurological function (such as motor learning capacities).

### Cell-specific IL-1β/IL-1R1 action for modulating the microglial displacement of GABAergic synapses

**Fig. 6:**
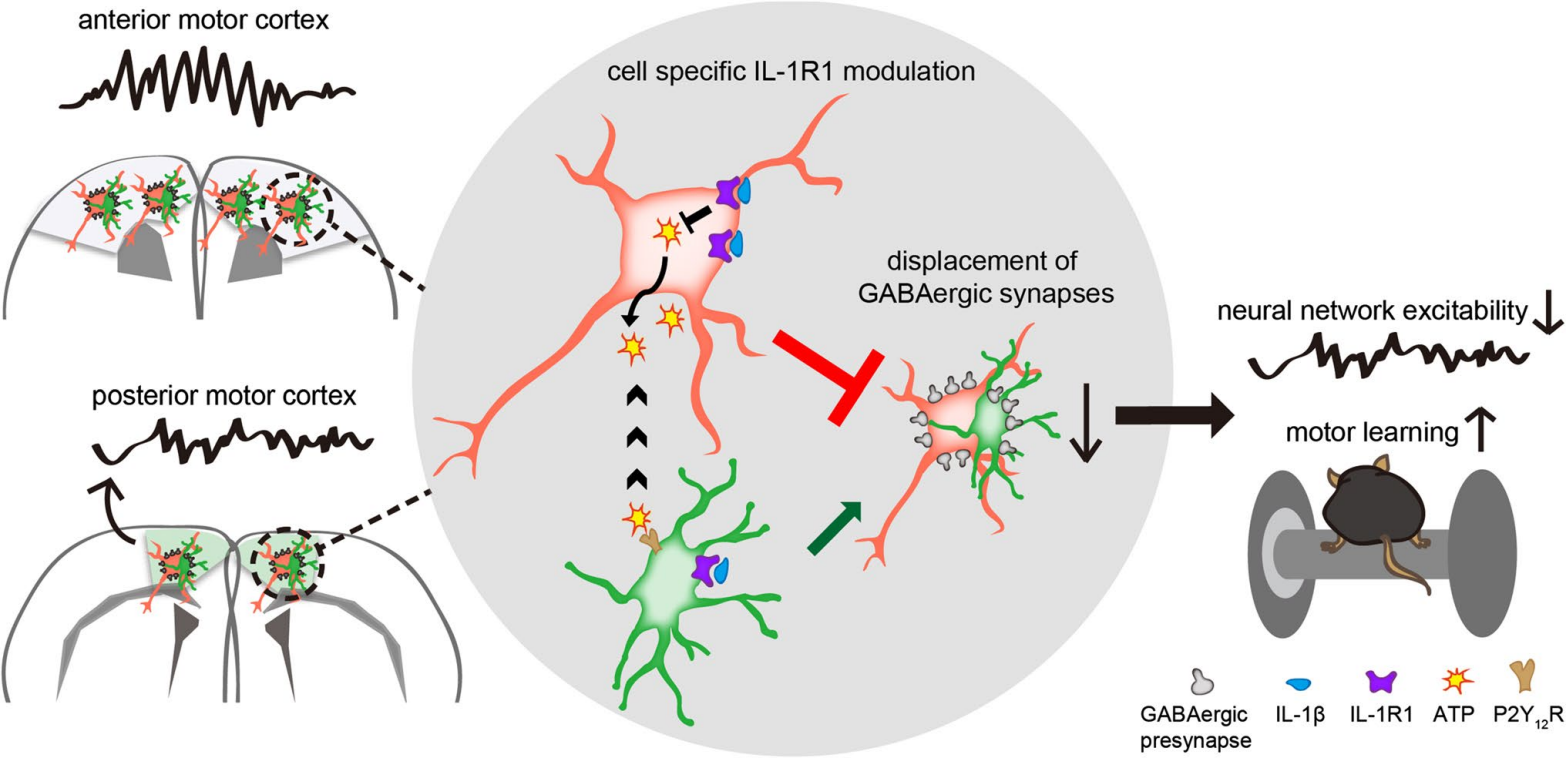
Graphical summary for the cell-specific role of IL-1R1 in the regulation of microglia-mediated synapse displacement, neural homeostasis and motor learning. IL-1β acts on IL-1R1 in neurons to prevent microglia from associating with neurons and synaptic displacement via reduction of ATP release and P2Y_12_R activation, thus modulating neural homeostasis and improving motor learning ability. However, IL-1R1 in microglia promotes synaptic displacement, which is overwhelmed by IL-1R1 in neurons. IL-1β/IL-1R1 finally contributes to the heterogeneity of microglial displacement that affects neural network homeostasis and motor learning ability

The regulatory role of IL-1R1 in synaptic displacement depends on cell type, which was determined by Cre-LoxP system. We found that only specific IL-1R1 deficits in glutamatergic neurons induced similar outcomes to IL-1R1 knockout or administration of IL-1Ra. In the co-culture system, we found a distance-dependent acceleration when microglia moved towards neuron soma, indicating that molecules released from neurons trigger microglial migration. The in vivo data indicate that ATP and P2Y_12_ receptors are also involved in the physiological microglial displacement of GABAergic synapses, however, this signaling serves downstream pathways of IL-1β and IL-1R1. Therefore, we speculate that IL-1β acting on IL-1R1 in glutamatergic neurons prevents their ATP production and inhibits microglial displacement, which subsequently tunes the neural network excitability and motor learning ability, but not the motor execution ability (Fig. 6).

Interestingly, we found that IL-1R1 on microglia induced opposite effects, while no obvious changes were observed during IL-1R1 deficits in GABAergic neurons. Specific microglial IL-1R1 deficits decreased microglial synaptic displacement, which aligns with the fact that synaptic displacement first requires microglial chemotaxis to the neuronal soma and IL-1R1 on microglia mediates chemotaxis of microglia [24]. The distinctive effects of IL-1R1 in different cells on synaptic displacement and motor learning suggest that IL-1R1 actions in glutamatergic neurons overwhelmed that in microglia under physiological conditions (Fig. 6). This fine-tuned modulation by microglia may locally and rapidly affect synaptic plasticity, neural networks, and neurological function.

In summary, this study revealed that microglia exhibited heterogeneity during the displacement of GABAergic synapses in different cortical regions under physiological conditions. IL-1β is critical for the regional heterogeneity of synaptic displacement by coordinating distinctive actions for neurons and microglia via IL-1R1, which impacts both neural network homeostasis and motor learning ability. This study provides a theoretical basis for elucidating the physiological role and mechanism of microglial synaptic displacement and provides a new paradigm by which microglia regulate synaptic plasticity and neurological function.

## Materials and methods

### Animals

This study used C57BL/6J mice and genetically modified mice with C57BL/6J mice, aged 8–10 weeks. Genetically modified mice included heterozygous GFP mice (*CX3CR1*^*GFP/*+^) expressing GFP under the promoter of the chemokine receptor (CX3CR1) (Jackson Laboratory, 005582), reporter mice expressing red fluorescence in a Cre enzyme-dependent manner (*Ai14*, Jackson Laboratory, 007914), mice with two LoxP sites inserted into the IL-1R1 gene *(IL-1R1*^*fl/f*^, Jackson Laboratory, 028398), mice with general IL-1R1 knockout (*IL-1R1*^*−/−*^, Jackson Laboratory, 003245), mice expressing the Cre enzyme under tamoxifen induction with CX3CR1 as a promoter (*CX3CR1*^*CreER/*+^, Jackson Laboratory, 021160), mice expressing the Cre enzyme with CaMKIIα as a promoter (*CaMKIIα*^*Cre*^, Jackson Laboratory, 005359), and mice expressing the Cre enzyme with VGAT as a promoter *(VGAT*^*Cre*^, Jackson Laboratory, 016962). Using Cre-LoxP technology, *IL-1R1*^*fl/f*l^ were crossed to *CX3CR1*^*CreER/*+^, *CaMKIIα*^*Cre*^, or *VGAT*^*Cre*^ to induce specific deficits of IL-1R1 on microglia, glutamatergic neurons, or GABAergic neurons, respectively. The primer sequences used for animal genotyping are shown in Table 1, and primers RB4082 and Reverse (*IL-1R1*^*fl/fl*^) were used to confirm whether IL-1R1 in the conditional knockout mice was systemically knocked out rather than conditionally knocked out in specific cells, as described previously (26930558). Animals were maintained under standard conditions (12 h light/dark cycle; 22–24 °C) with food and water ad libitum. The mice were handled according to the guidelines of the Animal Advisory Committee of Zhejiang University and the US National Institutes of Health Guidelines for the Care and Use of Laboratory Animals. All procedures were approved by the Animal Advisory Committee of Zhejiang University. During the experiments, we minimized the number of animals used and kept the suffering of the experimental animals to a minimum.

**Table 1:** Primer information

Animal	Primer type	Sequence (5′–3′)
*CX3CR1*^*GFP/*+^	Wild type forward	GTCTTCACGTTCGGTCTGGT
	Common	CCCAGACACTCGTTGTCCTT
	Mutant forward	CTCCCCCTGAACCTGAAAC
*Ai14*	Wild type forward	AAGGGAGCTGCAGTGGAGTA
	Wild type reverse	CCGAAAATCTGTGGGAAGTC
	Mutant reverse	GGCATTAAAGCAGCGTATCC
	Mutant forward	CTGTTCCTGTACGGCATGG
*IL-1R1*^*fl/fl*^	Forward	GAAAAGTGCTAGAACATCCTTTGAG
	Reverse	GTACCAATGGAGGCCAGAAG
	RB4082	CTTGTGTCCTATGGGTGTCC
*IL-1R1*^*−/−*^	Wild type forward	GAGTTACCCGAGGTCCAG
	Common	GAAGAAGCTCACGTTGTC
	Mutant forward	GCGAATGGGCTGACCGCT
*CX3CR1*^*CreER/*+^	Common	AAGACTCACGTGGACCTGCT
	Mutant reverse	CGGTTATTCAACTTGCACCA
	Wild type reverse	AGGATGTTGACTTCCGAGTTG
*CaMKIIα*^*Cre*^	Forward	TGCCCAAGAAGAAGAGGAA
	Reverse	TTGCAGGTACAGGAGGTAGTC
*VGAT*^*Cre*^	Forward	TCGATGCAACGAGTGATGAG
	Reverse	TCCATGAGTGAACGAACCTG

### Drug administration

For lateral ventricle injection, the mice were restrained in a stereotactic apparatus (512600, Stoelting, USA) under sodium pentobarbital anesthesia (50 mg/kg, i.p.; Abbott, North Chicago, IL, USA) while the cannula was embedded in the lateral ventricle (AP: − 0.4 mm, l: − 1.0 mm, V: − 2.5 mm). After 5–7 days of recovery, 1 μL of IL-1β, IL-1Ra or clopidogrel was injected into the lateral ventricle through the cannula for 5 min in freely moving mice, followed by a holding period of 5 min; injection dosage levels were as follows: 10 ng for IL-1β (prospect, USA), 100 ng for IL-1Ra (prospect, USA) and 50 μg for clopidogrel (Selleck, USA) as described previously [20, 21, 67].

We used the parenchyma injection for 3-consecutive-days rotarod test. Bilateral cannula was embedded in the anterior (AP: + 1.9 mm, l: ± 1.0 mm, V: − 0.5 mm) or posterior (AP: + 0.5 mm, l: ± 1.0 mm, V: − 0.5 mm) of the motor cortex. After 5–7 days of recovery, IL-1β (1 ng, 100 nl for each injection site in bilateral anterior motor cortex) or IL-1Ra (10 ng, 100 nl for each injection site in bilateral posterior motor cortex) was administrated for 3 min in freely moving mice followed by a holding period of 2 min. Mice received those reagents once per day, about 30 min before the first rotarod test. TRAM-34 (10 mg/kg, tocris, UK) was intraperitoneally injected 10 min before the brain parenchymal injection. *CX3CR1*^*CreER/*+^*;Ai14* mice and *CX3CR1*^*CreER/*+^*;IL-1R1*^*fl/fl*^ mice were intraperitoneally injected with 2 mg tamoxifen (10 mg/ml, Sigma, USA) for five consecutive days to induce red fluorescence or deficit of IL-1R1 in microglia as described previously [20].

Transcardial perfusion, behavior tests, or local field potential recording were performed about 30 min after drug administration.

### Immunofluorescence

The mice were anesthetized with sodium pentobarbital (50 mg/kg, i.p.) and transcardially perfused with 4% paraformaldehyde in PBS. Their brains were removed and immersed in 4% paraformaldehyde for post-fixation. After overnight storage at 4 °C, the 4% paraformaldehyde was replaced by a 30% sucrose solution for dehydration. Coronal sections at 30 μm were obtained using a freezing microtome (NX50, Thermo, USA). Sections were incubated in 0.5% Triton-PBS for 15 min at room temperature and blocked in 5% donkey serum for 1 h at room temperature. Slices were first incubated with primary antibodies overnight at 4 °C, washed three times in PBS at room temperature, incubated in secondary antibodies for 2 h at room temperature, and washed three times in PBS at room temperature. After being mounted with DAPI, 2/3 of the motor cortex layer was observed with a confocal laser scanning microscopy (SP8, Leica, Germany). 63 × objective (NA 1.40, oil) was applied with 0.5 μm z-step size for synapse imaging. Primary antibodies used in this study were as follows: goat anti-Iba-1, 1:500, Abcam, USA; rabbit anti-NeuN,1:500, Millipore, USA; mouse anti-VGAT conjugated with Oyster 550, 1:500, SYSY, Germany; Neuro Trace Nissl stain, 1:400 for 60 min at room temperature, Thermo Fisher Scientific, USA; rabbit anti-CaMKIIα, 1:300, Abcam, USA; rabbit-anti-GABA, 1:1000, Sigma Aldrich, USA. Secondary antibodies used in this study were as follows: anti-rabbit IgG-Alexa488, 1:500, Invitrogen, USA; anti-rabbit IgG-Alexa594, 1:500, Invitrogen, USA; anti-rabbit IgG-Alexa647, 1:500, Invitrogen, USA; anti-mouse lgG-Alexa488, 1:500, Invitrogen, USA; anti-mouse lgG-Alexa594, 1:500, Invitrogen, USA; anti-Goat lgG-Alexa488, 1:500, Invitrogen, USA; anti-Goat lgG-Alexa647, 1:500, Invitrogen, USA.

### Image analysis and 3D reconstruction

An extensive association was defined as when the microglia covered over one-quarter (25%) of the neuronal circumference in 2D confocal images and over 13.27% of the neuronal surface in 3D reconstruction images. The percentage of microglia extensively associated with neurons was calculated by dividing the number of microglia extensively associated with neurons over the total number of microglia in a micrograph field. We used the Imaris software (Version 9.5, bitplane A.G., Switzerland) to analyze the 3D reconstruction images. The "surface" function was used to render the cell or synaptic surface and reconstruct the three-dimensional structure, after which the contact area between the microglia and neurons was calculated using the "Surface contact area" plug-in. VGAT-labeled GABAergic presynapses, or VGAT and gephyrin co-labeled GABAergic synapses were identified using the "Spot" function, setting the XY diameter of the spot to 0.5 μm. The Z-axis was 1 μm. Using the "Filter" function to identify spots that were 0.5 μm away from the reconstructed cell surface, the number of spots was calculated as the number of GABAergic synapses around the cell bodies of neurons or microglia, which were closest to each other [68]. The 'Filter' function was used to filter out spots with a distance less than 0 μm from the microglia, which were GABAergic synapses engulfed by microglia. Neurons nearest to the center of the microglial soma were analyzed.

### In situ* hybridization*

The mice were anesthetized using sodium pentobarbital (50 mg/kg, i.p.) and transcardially perfused with 4% paraformaldehyde in PBS. Their brains were removed and immersed in 4% paraformaldehyde for post-fixation overnight at 4 °C. Gradient dehydration was performed by sequentially replacing 4% paraformaldehyde with 10%, 20%, 30% sucrose solution. Coronal sectioning at 10 μm was cut using a freezing microtome (NX50, Thermo). ACD RNAscope (for IL-1β mRNA) or Basescope (for IL-1R1 mRNA) kits were used for in situ hybridization staining. Once they were co-stained with immunofluorescence, the sections were subjected to immunostaining with primary antibody Iba-1 (1:500), NeuN (1:500) or GABA (1:1000), at 4 °C overnight, washed three times in PBS at room temperature, incubated in secondary antibodies for 2 h at room temperature, and again washed three times in PBS at room temperature. After being mounted with DAPI, layer 2/3 of the motor cortex was observed using a Leica SP8 confocal microscope.

### In vivo* two-photon surgery and calcium imaging*

*CX3CR1*^*CreER/*+^*;Ai14* mice were anesthetized with isoflurane and restrained in a stereotactic apparatus. A circular craniotomy (~ 3 mm in diameter) was performed over the right motor cortex (centered at 0.5 mm anterior and 1.5 mm lateral from bregma). To visualize neuronal activity in L2/3 pyramidal neurons, a pAOV-CaMKIIα-GCAMP6(s) virus (AAV2/9, 9.25 × 10^12^ vector genomes/ml, OBiO, Shanghai, China) was injected at 3 sites in L2/3 of the motor cortex using a glass pipette at an injection depth 200–300 μm below the cortical surface. Each site was injected with 500 nl of virus for 5 min; the glass pipette was left in the brain for an additional 5 min to avoid backflow. After the virus was injected, a glass window was implanted over the craniotomy, medical adhesive was used to stick the coverslip to the skull, and the edges of the cranial window were sealed with dental cement and dental adhesive resin cement. Tamoxifen was administered for 5 consecutive days beginning on the first day after surgery to induce red fluorescence expression in microglia. After the mice recovered for 2–3 weeks, calcium imaging was conducted using Olympus's FVMPE-RS deep imaging two-photon microscope with a two-photon pulsed femtosecond laser (690–1040 nm) and 20× objective. The excitation wavelength was 930 nm and continuous 1800-frame imaging was conducted for each field for 4 min at a depth of 150–200 μm below the cortical surface. Intracerebral administration of IL-1Ra was carried out through a soft catheter implanted in the lateral ventricles opposite the imaging window during the cranial window surgery. The resulting videos were analyzed using ImageJ software to calculate the fluorescence intensity and frequency of the neuronal calcium signals. The videos were corrected for focal plane displacements using TurboReg [69].

### In vivo local field potential surgery and recording

The in vivo local field potential experiments were performed as described previously [67]. After the 8-week-old mice were anesthetized with pentobarbital sodium (50 mg/kg, i.p.), they were restrained in a stereotactic apparatus. Electrodes (0.21 mm in diameter, a.m. systems, USA) were inserted into the anterior (AP: + 1.9 mm, l: − 1.0 mm, V: − 0.5 mm) and posterior (AP: + 0.5 mm, l: − 1.0 mm, V: − 0.5 mm) of the motor cortex, after which a bare wire was inserted into the cerebellar cortex to serve as grounding reference electrodes. For local field potential recording during IL-1β or IL-1Ra administration, electrodes (0.21 mm in diameter, a.m. systems, USA) were inserted into the anterior or posterior of the motor cortex, respectively, and a cannula was embedded in the lateral ventricle (AP: − 0.4 mm, l: + 1.0 mm, V: − 2.5 mm) for drug delivery. Mice were allowed to recover from surgery for 1 week before intracerebroventricular injection and local field potential recording. Neuronal signals were acquired using the Cerebus system (Version 6.04 BlackRock Microsystems, USA) at a sampling rate of 2 kHz. The power spectral density was analyzed using the Neuroexplorer software (Version 4.0) from 0.5 to 100 Hz.

### Western blot

The anterior motor cortex was rapidly dissected out and homogenized in RIPA buffer (pH 7.5, 20 mmol/l Tris–HCl, 150 mmol/l NaCl, 1 mmol/l EDTA, 1% Triton-X100, 0.5% sodium deoxycholate, 1 mmol/l PMSF and 10 μg/ml leupeptin). Protein samples (40 μg) were separated through SDS–polyacrylamide gel electrophoresis and transferred to a polyvinylidene fluoride membrane. The membrane was blocked with 5% skim milk in PBS for 1 h, and then membrane was incubated with primary antibodies against p-IκBα (1:500, ABclonal AP0707), IκBα (1:500, ABclonal A19714), p-p38 (1:500, ABclonal AP0526), or p38 (1:500, ABclonal A14401) overnight at 4 °C. Secondary antibody against rabbit (IRDye 800-coupled, 1:10,000) was incubated for 2 h at room temperature. Blots were visualized with the Odyssey infrared imaging system (LI-COR Biosciences) and analyzed with the Odyssey software. The measured values were compared with the mean values of the control group to obtain relative optical density values.

### Quantitative real-time polymerase chain reaction (qPCR)

The qPCR was modified with reference to previous methods [70]. Total RNA was isolated from flash-frozen whole brain samples using the MiniBEST Universal RNA Extraction Kit (TaKaRa#9767). RNA was isolated with DNase to avoid genomic DNA contamination. Individual samples shown were isolated from the anterior or posterior motor cortex of C57 mice. 1 μg of RNA was converted to cDNA with the PrimeScript™ RT reagent Kit with gDNA Eraser (TaKaRa#RR047). The mRNA levels were calculated by MonAmp™ SYBR Green qPCR Mix (Monad #MQ10101). The cDNA, fluorescently labeled primers, and kit reaction solution were added to the PCR tube, diluted appropriately, and then placed into a PCR instrument for thermal cycling, which consisted of three stages: denaturation, annealing, and extension. During the elongation phase, the fluorescence signal was monitored in real-time and the data were recorded. Data are normalized to β-actin mRNA level. The primer sequences used are shown in Table 2.

**Table 2:** Primer information for qPCR

Gene	Forward (5′–3′)	Reverse (5′–3′)
*IL-1B*	AAAGCTTGGTGATGTCTGGTC	GGACATGGAGAACACCACTTG
*BACT*	TGGCACCCAGCACAATGAA	CTAAGTCATAGTCCGCCTAGAAGCA

### Open field test

Mice aged 8–12 weeks were subjected to open field tests, which were performed from 10 a.m. to 5 p.m. Mice were allowed to acclimate to the test room for at least 30 min before the experiments began. A clear plexiglass box (45 × 45 × 45 cm) was used for the open field test. After administering IL-1β and IL-1Ra to the anterior and posterior motor cortex, respectively, the mice were placed in the center of the chamber while their movements were recorded and analyzed by automatic video tracking (ANY-maze 4.99) for 30 min. Locomotor activity was evaluated as the distance and average speed traveled per 10 min.

### Home cage test

Mice aged 8–12 weeks were subjected to home cage tests, which were performed from 10 a.m. to 5 p.m. Mice were allowed to acclimate to the test room for at least 30 min before the experiments began. After administering IL-1β and IL-1Ra to the anterior and posterior motor cortex, respectively, the mice were placed in a home cage equipped with a camera (AI Homecage XT, VanBi, Shanghai), which recorded their activity. Locomotor behaviors were evaluated for 2 h.

### Rotarod test

The accelerating rotarod test for motor learning ability was modified based on methods described previously [71–73]. Mice aged 8–12 weeks were subjected to rotarod tests, which were performed from 10 a.m. to 5 p.m. Mice were allowed to acclimate to the test room for at least 30 min before the experiments began and were then placed on a rotating cylinder (6 cm × 3 cm, YLS-4C, Zhenghua Biologic, China). The cylinder accelerated from 5 r.p.m. to 40 r.p.m. in 2 min, and whether or not the mouse fell from the rotarod was recorded. The maximum observation time was 5 min; if the mouse did not fall within 5 min, the experiment ended and the latency was recorded as 5 min. Each mouse was tested 6 times trials per day for 3 consecutive days, and the interval between each trial was at least 12 min. The excreta of mice were cleaned out after each trial and wiped with 75% alcohol to eliminate the influence of odor.

### Co-culture of primary microglia and primary neurons

The culture of primary neurons was performed as described previously [74]. Primary cortical neurons were obtained from 14–18-day-old embryos of pregnant C57BL6/J mice. The dissected cortex was digested with 0.25% trypsin (Invitrogen, USA). Approximately 10^5^ cells/cm^2^ were seeded onto poly-L-lysine (Sigma, USA) coated glass-bottom dishes (Cellvis, USA) for live-cell imaging. The neurons were grown in Neurobasal Plus medium (Invitrogen, USA) supplemented with 2% B27 Plus Supplement (Invitrogen, USA) and 0.25% GlutaMAX Supplement (Invitrogen, USA). Cultures were maintained for 8–12 d before treatment and half of the culture medium was replaced after 3 days.

For the culture of primary microglia, mouse brain mixed glial cells were prepared from the whole brains of 1–3 day-old postnatal *CX3CR1*^*GFP/*+^ mice and dissociated with mild mechanical trituration. Cells were seeded in cell culture bottles (75 cm^2^) precoated with poly-L-lysine at a density of 50,000 cells/cm^2^. The DMEM culture medium (Invitrogen, USA) was supplemented with 10% fetal bovine serum, 1% penicillin/streptomycin (all from Gibco, USA), while the media was added to reach a volume of 15 ml in the flasks. The culture medium was changed the next day to remove cell debris, after which it was changed every 5 days. To collect microglia, the flasks were vigorously tapped on the benchtop and the floating cells were collected. The culture medium of primary neurons was aspirated and replaced by 2 ml primary microglia for co-culture. Confocal pictures were taken after IL-1Ra administration (500 ng/ml) in the co-culture. The purity of neurons analyzed via immunofluorescence staining of NeuN was 96.30 ± 1.52% when microglia were excluded.

### Microdialysis

We anesthetized 8–10-week-old *CaMKIIα*^*Cre*^*;IL-1R1*^*fl/fl*^ mice and their littermates, *IL-1R1*^*fl/fl*^ mice, using sodium pentobarbital, after which they were restrained in a stereotaxic apparatus. A microdialysis guide cannula (MAB 10. 8. IC, Microbiotech, Sweden) was buried in the posterior motor cortex (AP: + 0.5 mm, l: − 1.0 mm, V: − 0.5 mm). The mice were given 3–4 weeks to recover from surgery, and then the extracellular fluid in the posterior motor cortex was collected using a Microbiotech microdialysis system. The ATP content was detected using high-performance liquid chromatography. The sample collection system was washed with ddH_2_O at 3 μl/min for 2 h, and then with artificial cerebrospinal fluid at 2 μl/min for 1 h. One hour after a microdialysis probe (MAB 10. 8. 1. PES, Microbiotech, Sweden) was inserted into the posterior motor cortex for equilibrium, cerebrospinal fluid was collected from the posterior motor cortex within 30 min. The ATP content in the posterior motor cortex was calibrated according to the recovery rates measured with standard substances.

### Statistical analysis

Statistical data are presented as mean ± standard error (mean ± SEM). Simple linear regression was used for the analysis of the correlation of 2D confocal images and 3D-reconstruction images. When the data fit a normal distribution, comparisons between two groups were analyzed using a Student’s *t* test, while comparisons between multiple groups were performed using one-way ANOVA combined with Bonferroni or Tukey’s multiple comparison test, according to the case number in compared groups, or two-way ANOVA combined with Bonferroni’s multiple comparison test. When the data did not follow a normal distribution, nonparametric tests, including Mann–Whitney test (comparisons between two groups), Kruskal–Wallis test (comparisons between multiple groups), Friedman test (comparisons between multiple groups with repeated measures) post hoc Dunn’s test were used. Regarding the quantification of 3D or calcium analysis base on cells (such as the number of GABAergic synapses, contact area, surface area, spike frequency et al.), the independent replicate in these experiments is the animal and cells within the same animal are not independent. According to the research of Yu et al. [75] and Lazic et al. [76], the most appropriate statistic is linear mixed modelling or generalized linear mixed modelling (depending on whether the data is normally distributed). So, we used a generalized linear model (normal distribution) or a generalized linear mixed model (none-normal distribution) to analyze these independent cells to avoid false positive. Regarding the comparison of the proportion of association, behavior phenotype and mRNA et al., statistical analysis was done considering N of mice, which were usually taken as independent replicates, Student's *t* test, one-way or two-way ANOVA, or nonparametric tests was applied. For all analyses, the tests were two-sided and *P* < 0.05 was considered statistically significant. The exact description of statistics and groups compared in each figure is seen in Table 3.

**Table 3:** Statistical data

Figure	Analysis	*N*
Figure 1b	One-way ANOVA with post hoc Tukey's test: *F* = 5.209, *P* < 0.001. M1/2–1 versus M1/2–3: *P* = 0.0411, S1/2–1 versus S1/2–4: *P* = 0.0351, S1/2–2 versus S1/2–4: *P* = 0.0224	*n* = 5 mice in each group
Figure 1e	Generalized linear mixed model with post hoc Bonferroni's test: *F*_3254_ = 14.262, *P* < 0.001. Anterior motor versus posterior motor: *P* < 0.001, anterior somatosensory versus posterior somatosensory: *P* < 0.001	Anterior motor: *n* = 76 cells, posterior motor: *n* = 70 cells, anterior somatosensory: *n* = 60 cells, posterior somatosensory: *n* = 52 cells. From 5 mice
Figure 1g	Generalized linear mixed model with post hoc Bonferroni's test: *F*_4, 372_ = 75.713, *P* < 0.001. Column factor: *F*_3, 372_ = 5.216, *P* = 0.002. Row factor: *F*_1, 372_ = 221.03, *P* < 0.001; anterior motor versus posterior motor: *P* = 0.04, anterior somatosensory versus posterior somatosensory: *P* = 0.014; comparison among “no association” groups in four regions: *P* = 0.174; comparison among “association” groups in four regions: *P* = 0.082	Anterior motor: *n* = 91 cells, posterior motor: *n* = 96 cells, anterior somatosensory: *n* = 100 cells, posterior somatosensory: *n* = 90 cells. From 5 mice
Figure 1h	Generalized linear mixed model with post hoc Bonferroni's test: *F*_4, 372_ = 70.859, *P* < 0.001. Column factor: *F*_3, 372_ = 7.482, *P* < 0.001. Row factor: *F*_1, 372_ = 188.601, *P* < 0.001; anterior motor versus posterior motor: *P* = 0.019, anterior somatosensory versus posterior somatosensory: *P* = 0.001; comparison among “no association” groups in four regions, *P* = 0.172; comparison among “association” groups in four regions: *P* = 0.357	
Figure 1i	Generalized Linear mixed model: *F*_4, 372_ = 0.170, *P* = 0.954. Column factor: *F*_3, 372_ = 0.216, *P* = 0.885. Row factor: *F*_1, 372_ = 0.047, *P* = 0.828; anterior motor versus posterior motor: *P* = 1, anterior somatosensory versus posterior somatosensory: *P* = 1	
Figure 1j	Generalized Linear mixed model: *F*_4, 372_ = 1.918, *P* = 0.107. Column factor: *F*_3, 372_ = 7.482, *P* = 0.096. Row factor: *F*_1, 372_ = , *P* = 0.92	
Figure 2b	Paired *t* test: *t*_6_ = 5.579, *P* = 0.0014	*n* = 7 mice in each group
Figure 2c	Simple linear regression: *F* = 16.16, *P* = 0.0017, *R*^2^ = 0.5738	*n* = 14 from 7 mice
Figure 2d	Paired *t* test: *t*_5_ = 7.686, *P* = 0.0006	*n* = 6 mice in each group
Figure 2f	One-way ANOVA with post hoc Tukey's test: *F* = 17.03, *P* < 0.0001. Anterior Vehicle versus anterior IL-1β: *P* = 0.0022, anterior Vehicle versus posterior Vehicle: *P* = 0.0005, posterior Vehicle versus posterior IL-1β: *P* = 0.3314, anterior IL-1β versus posterior IL-1β: *P* = 0.1085	*n* = 5 mice in each group
Figure 2g	Generalized linear mixed model with post hoc Bonferroni test: *F*_4, 371_ = 172.586, *P* < 0.001. Column factor: *F*_3, 371_ = 6.147, *P* < 0.001. Row factor: *F*_1, 371_ = 556.034, *P* < 0.001; anterior Vehicle versus anterior IL-1β: *P* = 0.005, anterior Vehicle versus poterior Vehicle: *P* = 0.003, poterior Vehicle versus poterior IL-1β: *P* = 1, anterior IL-1β versus poterior IL-1β: *P* = 1	Anterior vehicle: *n* = 99 cells from 5 mice, posterior Vehicle: *n* = 96 cells from 5 mice, anterior IL-1β: *n* = 89 cells from 5 mice, posterior IL-1β: *n* = 92 cells from 5 mice
Figure 2i	One-way ANOVA with post hoc Tukey's test: *F* = 15.84, *P* < 0.0001. Anterior Vehicle versus anterior IL-1Ra: *P* = 0.2752, anterior Vehicle versus posterior Vehicle: *P* = 0.0094, posterior Vehicle versus posterior IL-1Ra: *P* = 0.0001, anterior IL-1Ra versus posterior IL-1Ra: *P* = 0.9841	*n* = 4 mice in each group
Figure 2j	Generalized linear mixed model with post hoc Bonferroni test: *F*_4, 401_ = 232.058, *P* < 0.001. Column factor: *F*_3, 401_ = 4.203, *P* = 0.006. Row factor: *F*_1, 401_ = 825.864, *P* < 0.001; anterior Vehicle versus anterior IL-1Ra: *P* = 1, anterior Vehicle versus posterior Vehicle: *P* = 0.016, posterior Vehicle versus posterior IL-1Ra: *P* = 0.024, anterior IL-1Ra versus posterior IL-1Ra: *P* = 1	Anterior vehicle: *n* = 100 cells from 4 mice, posterior Vehicle: *n* = 106 cells from 4 mice, anterior IL-1Ra: *n* = 107 cells from 4 mice, posterior IL-1Ra: *n* = 93 cells from 4 mice
Figure 2l	One-way ANOVA with post hoc Bonferroni's test: *F* = 12.35, *P* = 0.0003. Anterior WT versus anterior *IL-1R1*^*−/−*^: *P* = 0.8050, anterior WT versus posterior WT: *P* = 0.0071, posterior WT versus posterior *IL-1R1*^*−/−*^: *P* = 0.0031, anterior *IL-1R1*^*−/−*^ versus posterior *IL-1R1*^*−/*−^: *P* = 0.9923	Anterior WT: *n* = 4 mice, posterior WT: *n* = 4 mice, anterior *IL-1R1*^*−/−*^: *n* = 5 mice, posterior *IL-1R1*^*−/−*^: *n* = 5 mice
Figure 2m	Generalized linear mixed model with post hoc Bonferroni's test: *F*_4, 383_ = 131.725, *P* < 0.001. Column factor: *F*_3, 383_ = 4.436, *P* = 0.004. Row factor: *F*_1, 383_ = 457.713, *P* < 0.001; anterior WT versus anterior *IL-1R1*^*−/−*^: *P* = 1, anterior WT versus posterior WT: *P* = 0.017, posterior WT versus posterior *IL-1R1*^*−/−*^: *P* = 0.009, anterior *IL-1R1*^*−/−*^ versus posterior *IL-1R1*^*−/−*^: *P* = 1	Anterior WT: *n* = 87 cells from 4 mice, posterior WT: *n* = 93 cells from 4 mice, anterior *IL-1R1*^*−/−*^: *n* = 102 cells from 5 mice, posterior *IL-1R1*^*−/−*^: *n* = 106 cells from 5 mice
Figure 3c	Mann–Whitney test: *U* = 0, *P* = 0.0159	CaMKIIα: *n* = 4 mice, GABA: *n* = 5 mice
Figure 3g	Generalized linear mixed model: *F* = 20.433, *P* < 0.001	No association: *n* = 53 cells, association: *n* = 41 cells. From 5 mice
Figure 3h	Generalized linear mixed model: *F* = 0.07, *P* = 0.792	
Figure 3m	Friedman test post hoc Dunn's test: Friedman statistic = 10.63, *P* = 0.0139. Base versus Pre: *P > *0.9999, Base versus Association: *P* = 0.0427, Base versus Post: *P > *0.9999	*n* = 7 cells from 3 mice in each group
Figure 3n	Friedman test: Friedman statistic = 3.514, *P* = 0.3189	
Figure 3p	Two-way ANOVA test: Row Factor:* F* = 7.054,* P* < 0.0001, Column Factor (anterior vs. posterior): *F* = 37.72, *P* < 0.0001	*n* = 3 mice in each group
Figure 3r	Two-way ANOVA test: Row Factor:* F* = 2.987, *P* < 0.0001, Column Factor (anterior-Vehicle vs. anterior-IL-1β): *F* = 1226, *P* < 0.0001	*n* = 5 mice in each group
Figure 3t	Two-way ANOVA test: Row Factor:* F* = 0.2999, *P > *0.9999, Column Factor (posterior-Vehicle vs. posterior-IL-1Ra): *F*_1*,*1430_ = 194.5, *P* < 0.0001	*n* = 6 mice in each group
Figure 3u	Repeated measures two-way ANOVA test: Time Factor:* F* = 2.962, *P* < 0.0001, Column Factor (anterior-Vehicle vs. anterior-IL-1β): *F*_1*,*19_ = 4.407, *P* = 0.0494	anterior-Vehicle: *n* = 10 mice, anterior-IL-1β: *n* = 11 mice
Figure 3v	Repeated measures two-way ANOVA with post hoc Tukey's test: Time Factor:* F* = 14.16,* P* < 0.0001, Column Factor: *F*_2*,*24_ = 6.266, *P* = 0.0434; posterior-Vehicle versus poserior-IL-1Ra: *P* = 0.0434, posterior-Vehicle versus poserior-IL-1Ra + TRAM-34: *P* = 0.6877, poserior-IL-1Ra versus poserior-IL-1Ra + TRAM-34: *P* = 0.0065	*n* = 9 mice in each group
Figure 3x	One-way ANOVA with post hoc Tukey's test: *F*_2*,*12_ = 8.464, *P* = 0.0051. Vehicle versus IL-1Ra: *P* = 0.0173, Vehicle versus IL-1Ra + TRAM-34: *P* = 0.8528, IL-1Ra versus IL-1Ra + TRAM-34: *P* = 0.0066	*n* = 5 mice in each group
Figure 3y	Generalized linear mixed model with post hoc Bonferroni's test: *F*_3,294_ = 235.319, *P* < 0.001. Column factor: *F*_2,294_ = 6.412, *P* = 0.002. Row factor: *F*_1,294_ = 613.243, *P* < 0.001; Vehicle versus IL-1Ra: *P* = 0.014, Vehicle versus IL-1Ra + TRAM-34: *P* = 0.495, IL-1Ra versus IL-1Ra + TRAM-34: *P* = 0.003	Vehicle: *n* = 98 cells from 5 mice, IL-1Ra: *n* = 106 cells from 5 mice, IL-1Ra + TRAM-34: *n* = 94 cells from 5 mice
Figure 4b	One-way ANOVA with post hoc Tukey's test: *F*_3,12_ = 8.842, *P* = 0.0023. anterior *IL-1R1*^*fl/fl*^ versus anterior *CX3CR1*^*CreER/*+^*;IL-1R1*^*fl/fl*^: *P* = 0.0224, anterior *IL-1R1*^*fl/fl*^ versus posterior *IL-1R1*^*fl/fl*^: *P* = 0.0096, posterior *IL-1R1*^*fl/fl*^ versus posterior *CX3CR1*^*CreER/*+^*;IL-1R1*^*fl/fl*^: *P* = 0.8101, anterior *CX3CR1*^*CreER/*+^*;IL-1R1*^*fl/fl*^ versus posterior *CX3CR1*^*CreER/*+^*;IL-1R1*^*fl/fl*^: *P* = 0.5379	*n* = 4 mice in each group
Figure 4c	Generalized linear mixed model with post hoc Bonferroni's test: *F*_4,390_ = 225.598, *P* < 0.001. Column factor: *F*_3,390_ = 7.342, *P* < 0.001. Row factor: *F*_1,390_ = 792.276, *P* < 0.001; anterior *IL-1R1*^*fl/fl*^ versus anterior *CX3CR1*^*CreER/*+^*;IL-1R1*^*fl/fl*^: *P* = 0.001, anterior *IL-1R1*^*fl/fl*^ versus posterior *IL-1R1*^*fl/fl*^: *P* = 0.002, posterior *IL-1R1*^*fl/fl*^ versus posterior *CX3CR1*^*CreER/*+^*;IL-1R1*^*fl/fl*^: *P* = 1, anterior *CX3CR1*^*CreER/*+^*;IL-1R1*^*fl/fl*^ versus posterior *CX3CR1*^*CreER/*+^*;IL-1R1*^*fl/fl*^: *P* = 1	Anterior *IL-1R1*^*fl/fl*^: *n* = 107 cells from 4 mice, posterior *IL-1R1*^*fl/fl*^: *n* = 94 cells from 4 mice, anterior *CX3CR1*^*CreER/*+^*;IL-1R1*^*fl/fl*^: *n* = 104 cells from 4 mice, posterior *CX3CR1*^*CreER/*+^*;IL-1R1*^*fl/fl*^: *n* = 90 cells from 4 mice
Figure 4e	Two-way ANOVA test: Row Factor:* F* = 34.42, *P* < 0.0001, Column Factor (*IL-1R1*^*fl/fl*^ vs. *CX3CR1*^*CreER/*+^*;IL-1R1*^*fl/fl*^): *F*_1*,*1287_ = 15.48, *P* < 0.0001	*IL-1R1*^*fl/fl*^: *n* = 5 mice, *CX3CR1*^*CreER/*+^*;IL-1R1*^*fl/fl*^: *n* = 6 mice
Figure 4f	Repeated measures two-way ANOVA test: Time Factor:* F* = 16.01,* P* < 0.0001, Column Factor (*IL-1R1*^*fl/fl*^ vs. *CX3CR1*^*CreER/*+^*;IL-1R1*^*fl/fl*^): *F*_1*,*22_ = 4.431, *P* = 0.0469	*n* = 12 mice in each group
Figure 5b	One-way ANOVA with post hoc Tukey's test: *F*_3,12_ = 20, *P* < 0.001. anterior *IL-1R1*^*fl/fl*^ versus anterior *CaMKII*α^*Cre*^*;IL-1R1*^*fl/fl*^: *P* = 0.23, anterior *IL-1R1*^*fl/fl*^ versus posterior *IL-1R1*^*fl/fl*^: *P* = 0.004, posterior *IL-1R1*^*fl/fl*^ versus posterior *CaMKIIα*^*Cre*^*;IL-1R1*^*fl/fl*^: *P* < 0.001, anterior *CaMKIIα*^*Cre*^*;IL-1R1*^*fl/fl*^ versus posterior *CaMKIIα*^*Cre*^*;IL-1R1*^*fl/fl*^: *P* = 0.99	*n* = 4 mice in each group
Figure 5c	Generalized linear mixed model with post hoc Bonferroni's test: *F*_4,399_ = 162.967, *P* < 0.001. Column factor: *F*_3,399_ = 3.125, *P* = 0.026. Row factor: *F*_1,399_ = 594.975, *P* < 0.001; anterior *IL-1R1*^*fl/fl*^ versus anterior *CaMKII*α^*Cre*^*;IL-1R1*^*fl/fl*^: *P* = 1, anterior *IL-1R1*^*fl/fl*^ versus posterior *IL-1R1*^*fl/fl*^: *P* = 0.048, posterior *IL-1R1*^*fl/fl*^ versus posterior *CaMKIIα*^*Cre*^*;IL-1R1*^*fl/fl*^: *P* = 0.007, anterior *CaMKIIα*^*Cre*^*;IL-1R1*^*fl/fl*^ versus posterior *CaMKIIα*^*Cre*^*;IL-1R1*^*fl/fl*^: *P* = 1	Anterior *IL-1R1*^*fl/fl*^: *n* = 95 cells from 4 mice, posterior *IL-1R1*^*fl/fl*^: *n* = 94 cells from 4 mice, anterior *CaMKIIα*^*Cre*^*;IL-1R1*^*fl/fl*^: *n* = 109 cells from 4 mice, posterior *CaMKIIα*^*Cre*^*;IL-1R1*^*fl/fl*^: *n* = 106 cells from 4 mice
Figure 5e	Two-way ANOVA test: Row Factor:* F* = 9.995,* P* < 0.0001, Column Factor (*IL-1R1*^*fl/fl*^ vs. *CaMKIIα*^*Cre*^*;IL-1R1*^*fl/fl*^): *F*_1*,*1144_ = 209, *P* < 0.0001	*n* = 5 mice in each group
Figure 5f	Repeated measures two-way ANOVA test: Time Factor:* F* = 6.043, *P* < 0.0001, Column Factor (*IL-1R1*^*fl/fl*^ vs. *CaMKIIα*^*Cre*^*;IL-1R1*^*fl/fl*^): *F*_1*,*17_ = 5.242, *P* = 0.0351	*IL-1R1*^*fl/fl*^: *n* = 9 mice, *CaMKIIα*^*Cre*^*;IL-1R1*^*fl/fl*^: *n* = 10 mice
Figure 5h	Unpaired *t* test: *t*_7_ = 7.103, *P* = 0.0002	Vehicle *n* = 4 mice, Clopidogrel: *n* = 5 mice
Figure 5i	Generalized linear mixed model post hoc Bonferroni's test: *F*_2,255_ = 252.182, *P* < 0.001. Column factor: *F*_1,255_ = 4.602, *P* = 0.033. Row factor: *F*_1,255_ = 480.411, *P* < 0.001; Vehicle versus Clopidogrel, *P* = 0.033	Vehicle *n* = 130 cells from 4 mice, Clopidogrel: *n* = 128 cells from 5 mice
Figure 5j	Unpaired *t* test: *t*_9_ = 2.479, *P* = 0.0356	*IL-1R1*^*fl/fl*^: *n* = 5 mice, *CaMKIIα*^*Cre*^*;IL-1R1*^*fl/fl*^: *n* = 6 mice
Fig. S1A	Simple linear regression: *Y* = 0.545 × *X* − 0.355, *P* < 0.0001, *R*^2^ = 0.774	*n* = 119 cells from 5 mice
Fig. S1B	One-way ANOVA with post hoc Tukey's test: *F* = 15.68, *P* < 0.0001. anterior motor versus posterior motor: *P* = 0.002, anterior somatosensory versus posterior somatosensory: *P* = 0.0005	*n* = 5 mice in each group
Fig. S1C	Generalized linear mixed model post hoc Bonferroni's test: *F*_3,218_ = 2.307, *P* = 0.078	Anterior motor: *n* = 58 cells, posterior motor: *n* = 41 cells, anterior somatosensory: *n* = 60 cells, posterior somatosensory: *n* = 64 cells. From 5 mice
Fig. S1D	Generalized linear mixed model post hoc Bonferroni's test: *F*_3,218_ = 1.698, *P* = 0.168	Anterior motor: *n* = 58 cells, posterior motor: *n* = 41 cells, anterior somatosensory: *n* = 60 cells, posterior somatosensory: *n* = 64 cells. From 5 mice
Fig. S2A	One-way ANOVA test: *F* = 1.129, *P* = 0.3403	*n* = 5 mice in each group
Fig. S2B	One-way ANOVA test: *F* = 1.181, *P* = 0.3483	*n* = 5 mice in each group
Fig. S2C	One-way ANOVA test: *F* = 0.8381, *P* = 0.4953	*n* = 4 mice in each group
Fig. S2D	One-way ANOVA test: *F* = 0.7128, *P* = 0.5604	Anterior WT: *n* = 4 mice, posterior WT: *n* = 4 mice, anterior *IL-1R1*^*−/−*^: *n* = 5 mice, posterior *IL-1R1*^*−/−*^: *n* = 5 mice
Fig. S2E	One-way ANOVA test: *F* = 1.441, *P* = 0.2748	*n* = 5 mice in each group
Fig. S2F	One-way ANOVA test: *F* = 0.4493, *P* = 0.7224	*n* = 4 mice in each group
Fig. S2G	One-way ANOVA test: *F* = 1.377, *P* = 0.2971	*n* = 4 mice in each group
Fig. S2H	Unpaired *t* test: *t*_7_ = 0.04248, *P* = 0.9673	Vehicle: *n* = 4 mice, Clopidogrel: *n* = 5 mice
Fig. S3B	Generalized linear mixed model: *F*_3,97_ = 0.027, *P* = 0.994	Anterior motor: *n* = 34 cells, posterior motor: *n* = 16 cells, anterior somatosensory: *n* = 16 cells, posterior somatosensory: *n* = 35 cells. From 3 mice
Fig. S3C	Generalized linear mixed model with post hoc Bonferroni's test: *F*_4, 375_ = 185.938, *P* < 0.001. Column factor: *F*_3, 375_ = 11.445, *P* < 0.001. Row factor: *F*_1, 375_ = 531.653, *P* < 0.001; anterior motor versus posterior motor: *P* < 0.001, anterior somatosensory versus posterior somatosensory: *P* < 0.001	Anterior motor: *n* = 86 cells, posterior motor: *n* = 102 cells, anterior somatosensory: *n* = 107 cells, posterior somatosensory: *n* = 85 cells. From 4 mice
Fig. S3D	Generalized linear mixed model: *F*_4, 259_ = 2.135, *P* = 0.077. Column factor: *F*_3, 259_ = 1.613, *P* = 0.187. Row factor: *F*_1, 259_ = 3.652, *P* = 0.057	Anterior motor: *n* = 64 cells, posterior motor: *n* = 68 cells, anterior somatosensory: *n* = 70 cells, posterior somatosensory: *n* = 62 cells. From 3 mice
Fig. S4C	Two-way ANOVA with post hoc Tukey's test: *F* = 1.917, *P* = 0.0063. Immobile-Vehicle versus Immobile-IL-1Ra: *P* = 0.003, Away-Vehicle versus Away-IL-1Ra: *P* = 0.0024, Towards-Vehicle versus Towards-IL-1Ra: *P* < 0.0001	*n* = 9 neurons in each group
Fig. S4D	Two-way ANOVA with post hoc Bonferroni's test: *F* = 5.540, *P* = 0.0002. Vehicle versus IL-1Ra: *P* < 0.0001	
Fig. S4E	Two-way ANOVA with post hoc Bonferroni's test: *F* = 5.171, *P* < 0.0001. Vehicle versus IL-1Ra: *P* < 0.0001	
Fig. S4G	Two-way ANOVA with post hoc Bonferroni's test: *F* = 32.17, *P* < 0.0001. Vehicle versus IL-1Ra: *P* < 0.0001	
Fig. S5B	Mann–Whitney test: *U* = 11, *P* = 0.8413	*n* = 5 mice in each group
Fig. S5D	Mann–Whitney test: *U* = 7, *P* = 0.3095	*n* = 5 mice in each group
Fig. S6B	Generalized linear mixed model with post hoc Bonferroni test: *F*_4, 390_ = 257.297, *P* < 0.001. Column factor: *F*_3, 390_ = 9.254, *P* < 0.001. Row factor: *F*_1, 390_ = 900.986, *P* < 0.001; anterior Vehicle versus anterior IL-1Ra: *P* = 0.592, anterior Vehicle versus posterior Vehicle: *P* < 0.001, posterior Vehicle versus posterior IL-1Ra: *P* < 0.001, anterior IL-1Ra versus posterior IL-1Ra: *P* = 0.157	Anterior vehicle: *n* = 95 cells from 4 mice, posterior Vehicle: *n* = 107 cells from 4 mice, anterior IL-1Ra: *n* = 98 cells from 4 mice, posterior IL-1Ra: *n* = 101 cells from 4 mice
Fig. S7A	Two-way ANOVA test: Row Factor:* F* = 3.526,* P* = 0.0399, Column Factor (anterior-Vehicle vs. anterior-IL-1β): *F*_1*,*36_ = 0.94, *P* = 0.3388	*n* = 7 mice in each group
Fig. S7B	Two-way ANOVA test: Row Factor:* F* = 3.578,* P* = 0.0383, Column Factor (anterior-Vehicle vs. anterior-IL-1β): *F*_1*,*36_ = 1.89, *P* = 0.1777	*n* = 7 mice in each group
Fig. S7C	Unpaired *t* test: *t*_12_ = 0.9568, *P* = 0.3575	*n* = 7 mice in each group
Fig. S7D	Unpaired *t* test: *t*_12_ = 0.958, *P* = 0.357	*n* = 7 mice in each group
Fig. S7E	Unpaired *t* test: *t*_12_ = 0.7276, *P* = 0.4808	*n* = 7 mice in each group
Fig. S7F	Unpaired *t* test: *t*_12_ = 0.7276, *P* = 0.4808	*n* = 7 mice in each group
Fig. S7G	Unpaired *t* test: *t*_12_ = 0.6571, *P* = 0.5235	*n* = 7 mice in each group
Fig. S7H	Unpaired *t* test: *t*_12_ = 0.6352, *P* = 0.5372	*n* = 7 mice in each group
Fig. S7I	Repeated measures two-way ANOVA test: Time Factor:* F* = 15.62, *P* < 0.0001, Column Factor (poserior-Vehicle vs. posterior-IL-1β): *F* = 0.1838, *P* = 0.6746	*n* = 8 mice in each group
Fig. S7J	Two-way ANOVA test: Row Factor:* F* = 1.725, *P* = 0.1926, Column Factor (posterior-Vehicle vs. posterior-IL-1Ra): *F*_1*,*36_ = 0.5757, *P* = 0.453	*n* = 7 mice in each group
Fig. S7K	Two-way ANOVA test: Row Factor:* F* = 0.6193, *P* = 0.5439, Column Factor (posterior-Vehicle vs. posterior-IL-1Ra): *F*_1*,*36_ = 0.01088, *P* = 0.9175	*n* = 7 mice in each group
Fig. S7L	Unpaired *t* test: *t*_12_ = 0.6418, *P* = 0.5331	*n* = 7 mice in each group
Fig. S7M	Unpaired *t* test: *t*_12_ = 0.6544, *P* = 0.5252	*n* = 7 mice in each group
Fig. S7N	Unpaired *t* test: *t*_12_ = 0.187, *P* = 0.8548	*n* = 7 mice in each group
Fig. S7O	Unpaired *t* test: *t*_12_ = 0.187, *P* = 0.8548	*n* = 7 mice in each group
Fig. S7P	Unpaired *t* test: *t*_12_ = 0.0075, *P* = 0.9942	*n* = 7 mice in each group
Fig. S7Q	Mann–Whitney test: *U* = 198, *P* = 0.535	*n* = 7 mice in each group
Fig. S7R	Repeated measures two-way ANOVA test: Time Factor:* F* = 6.468, *P* < 0.0001, Column Factor (anterior-Vehicle vs. anterior-IL-1Ra): *F*_1*,*15_ = 0.4164, *P* = 0.5285	anterior-Vehicle: *n* = 8 mice, anterior-IL-1Ra: *n* = 9 mice
Fig. S8C	Unpaired *t* test: *t*_4_ = 4.004, *P* = 0.0161	*n* = 3 mice in each group
Fig. S8E	Unpaired *t* test: *t*_4_ = 2.793, *P* = 0.0492	*n* = 3 mice in each group
Fig. S8G	Unpaired *t* test: *t*_4_ = 3.664, *P* = 0.0215	*n* = 3 mice in each group
Fig. S9B	Generalized linear mixed model with post hoc Bonferroni's test: *F*_4,370_ = 295.214, *P* < 0.001. Column factor: *F*_3,370_ = 8.696, *P* < 0.001. Row factor: *F*_1,370_ = 1022.914, *P* < 0.001; anterior *IL-1R1*^*fl/fl*^ versus anterior *CaMKII*α^*Cre*^*;IL-1R1*^*fl/fl*^: *P* = 1, anterior *IL-1R1*^*fl/fl*^ versus posterior *IL-1R1*^*fl/fl*^: *P* < 0.001, posterior *IL-1R1*^*fl/fl*^ versus posterior *CaMKIIα*^*Cre*^*;IL-1R1*^*fl/fl*^: *P* = 0.001, anterior *CaMKIIα*^*Cre*^*;IL-1R1*^*fl/fl*^ versus posterior *CaMKIIα*^*Cre*^*;IL-1R1*^*fl/fl*^: *P* = 1	anterior *IL-1R1*^*fl/fl*^: *n* = 89 cells from 4 mice, posterior *IL-1R1*^*fl/fl*^: *n* = 96 cells from 4 mice, anterior *CaMKIIα*^*Cre*^*;IL-1R1*^*fl/fl*^: *n* = 93 cells from 4 mice, posterior *CaMKIIα*^*Cre*^*;IL-1R1*^*fl/fl*^: *n* = 97 cells from 4 mice
Fig. S10B	One-way ANOVA with post hoc Tukey's test: *F*_3,12_ = 12.53, *P* = 0.0005. anterior *IL-1R1*^*fl/fl*^ versus anterior *VGAT*^*Cre*^*;IL-1R1*^*fl/fl*^: *P* = 0.6564, anterior *IL-1R1*^*fl/fl*^ versus posterior *IL-1R1*^*fl/fl*^: *P* = 0.0089, posterior *IL-1R1*^*fl/fl*^ versus posterior *VGAT*^*Cre*^*;IL-1R1*^*fl/fl*^: *P* = 0.9235, anterior *VGAT*^*Cre*^*;IL-1R1*^*fl/fl*^ versus posterior *VGAT*^*Cre*+^*;IL-1R1*^*fl/fl*^: *P* = 0.0035	*n* = 4 mice in each group
Fig. S10C	One-way ANOVA test: *F*_3*,*12_ = 0.5691, *P* = 0.6459	*n* = 4 mice in each group

### Supplementary Information

Below is the link to the electronic supplementary material.

**Supplementary file 1** (AVI 37275 KB)

**Supplementary file 2** (AVI 32832 KB)

**Supplementary file 3** (AVI 17594 KB)

**Supplementary file 4** (AVI 13272 KB)

**Supplementary file 5** (AVI 9033 KB)

**Supplementary file 6** (AVI 18539 KB)

**Supplementary file 7** (DOCX 98922 KB)

## Data Availability

All data supporting the findings of this study are available within the paper and its Supplementary Information.

## References

[1] Butovsky O, Weiner HL (2018). Microglial signatures and their role in health and disease. Nat Rev Neurosci.

[2] Borst K, Dumas AA, Prinz M (2021). Microglia: immune and non-immune functions. Immunity.

[3] Hambardzumyan D, Gutmann DH, Kettenmann H (2016). The role of microglia and macrophages in glioma maintenance and progression. Nat Neurosci.

[4] Wang M, Jiang Y, Huang Z (2022). Loss of C9orf72 in microglia drives neuronal injury by enhancing synaptic pruning in aged and Alzheimer’s disease mice. Neurosci Bull.

[5] Zheng L, Wang Y, Shao B, Zhou H, Li X (2022). Multiple mild stimulations reduce membrane distribution of CX3CR1 promoted by annexin a1 in microglia to attenuate excessive dendritic spine pruning and cognitive deficits caused by a transient ischemic attack in mice. Neurosci Bull.

[6] Schafer DP, Lehrman EK, Kautzman AG, Koyama R, Mardinly AR (2012). Microglia sculpt postnatal neural circuits in an activity and complement-dependent manner. Neuron.

[7] Wu Y, Dissing-Olesen L, MacVicar BA, Stevens B (2015). Microglia: dynamic mediators of synapse development and plasticity. Trends Immunol.

[8] Cserep C, Posfai B, Denes A (2021). Shaping neuronal fate: functional heterogeneity of direct microglia–neuron interactions. Neuron.

[9] Paolicelli RC, Bolasco G, Pagani F, Maggi L, Scianni M (2011). Synaptic pruning by microglia is necessary for normal brain development. Science.

[10] Wu C, Yang L, Youngblood H, Liu TC, Duan R (2022). Microglial SIRPalpha deletion facilitates synapse loss in preclinical models of neurodegeneration. Neurosci Bull.

[11] Wang C, Yue H, Hu Z, Shen Y, Ma J (2020). Microglia mediate forgetting via complement-dependent synaptic elimination. Science.

[12] Wake H, Moorhouse AJ, Jinno S, Kohsaka S, Nabekura J (2009). Resting microglia directly monitor the functional state of synapses in vivo and determine the fate of ischemic terminals. J Neurosci.

[13] Salter MW, Stevens B (2017). Microglia emerge as central players in brain disease. Nat Med.

[14] Hong S, Beja-Glasser VF, Nfonoyim BM, Frouin A, Li S (2016). Complement and microglia mediate early synapse loss in Alzheimer mouse models. Science.

[15] Miyamoto A, Wake H, Ishikawa AW, Eto K, Shibata K (2016). Microglia contact induces synapse formation in developing somatosensory cortex. Nat Commun.

[16] Blinzinger K, Kreutzberg G (1968). Displacement of synaptic terminals from regenerating motoneurons by microglial cells. Z Zellforsch Mikrosk Anat.

[17] Chen Z, Jalabi W, Shpargel KB, Farabaugh KT, Dutta R (2012). Lipopolysaccharide-induced microglial activation and neuroprotection against experimental brain injury is independent of hematogenous TLR4. J Neurosci.

[18] Chen Z, Jalabi W, Hu W, Park HJ, Gale JT (2014). Microglial displacement of inhibitory synapses provides neuroprotection in the adult brain. Nat Commun.

[19] Trapp BD, Wujek JR, Criste GA, Jalabi W, Yin X (2007). Evidence for synaptic stripping by cortical microglia. Glia.

[20] Wan Y, Feng B, You Y, Yu J, Xu C (2020). Microglial displacement of GABAergic synapses is a protective event during complex febrile seizures. Cell Rep.

[21] Feng B, Tang Y, Chen B, Xu C, Wang Y (2016). Transient increase of interleukin-1beta after prolonged febrile seizures promotes adult epileptogenesis through long-lasting upregulating endocannabinoid signaling. Sci Rep.

[22] Prieto GA, Snigdha S, Baglietto-Vargas D, Smith ED, Berchtold NC (2015). Synapse-specific IL-1 receptor subunit reconfiguration augments vulnerability to IL-1beta in the aged hippocampus. Proc Natl Acad Sci USA.

[23] Xu C, Zhang S, Gong Y, Nao J, Shen Y (2021). Subicular caspase-1 contributes to pharmacoresistance in temporal lobe epilepsy. Ann Neurol.

[24] Ferreira R, Santos T, Cortes L, Cochaud S, Agasse F (2012). Neuropeptide Y inhibits interleukin-1 beta-induced microglia motility. J Neurochem.

[25] Matcovitch-Natan O, Winter DR, Giladi A, Vargas Aguilar S, Spinrad A (2016). Microglia development follows a stepwise program to regulate brain homeostasis. Science.

[26] Kettenmann H, Kirchhoff F, Verkhratsky A (2013). Microglia: new roles for the synaptic stripper. Neuron.

[27] Silvin A, Ginhoux F (2018). Microglia heterogeneity along a spatio-temporal axis: more questions than answers. Glia.

[28] De Biase LM, Schuebel KE, Fusfeld ZH, Jair K, Hawes IA (2017). Local cues establish and maintain region-specific phenotypes of basal ganglia microglia. Neuron.

[29] Liu YU, Ying Y, Li Y, Eyo UB, Chen T (2019). Neuronal network activity controls microglial process surveillance in awake mice via norepinephrine signaling. Nat Neurosci.

[30] Madry C, Kyrargyri V, Arancibia-Carcamo IL, Jolivet R, Kohsaka S (2018). Microglial ramification, surveillance, and interleukin-1beta release are regulated by the two-pore domain K(+) channel THIK-1. Neuron.

[31] Kubota Y, Karube F, Nomura M, Kawaguchi Y (2016). The diversity of cortical inhibitory synapses. Front Neural Circuits.

[32] Akash MS, Rehman K, Chen S (2013). IL-1Ra and its delivery strategies: inserting the association in perspective. Pharm Res.

[33] Spulber S, Bartfai T, Schultzberg M (2009). IL-1/IL-1ra balance in the brain revisited-evidence from transgenic mouse models. Brain Behav Immun.

[34] Chen G, Zhang Y, Li X, Zhao X, Ye Q (2017). Distinct inhibitory circuits orchestrate cortical beta and gamma band oscillations. Neuron.

[35] Salkoff DB, Zagha E, Yuzgec O, McCormick DA (2015). Synaptic mechanisms of tight spike synchrony at gamma frequency in cerebral cortex. J Neurosci.

[36] Nowak M, Zich C, Stagg CJ (2018). Motor cortical gamma oscillations: What have we learnt and where are we headed?. Curr Behav Neurosci Rep.

[37] Kawai R, Markman T, Poddar R, Ko R, Fantana AL (2015). Motor cortex is required for learning but not for executing a motor skill. Neuron.

[38] Peters AJ, Liu H, Komiyama T (2017). Learning in the rodent motor cortex. Annu Rev Neurosci.

[39] Peters AJ, Chen SX, Komiyama T (2014). Emergence of reproducible spatiotemporal activity during motor learning. Nature.

[40] Mailhot B, Christin M, Tessandier N, Sotoudeh C, Bretheau F (2020). Neuronal interleukin-1 receptors mediate pain in chronic inflammatory diseases. J Exp Med.

[41] Walsh JG, Muruve DA, Power C (2014). Inflammasomes in the CNS. Nat Rev Neurosci.

[42] Liu X, Nemeth DP, McKim DB, Zhu L, DiSabato DJ (2019). Cell-type-specific interleukin 1 receptor 1 signaling in the brain regulates distinct neuroimmune activities. Immunity.

[43] Nemeth DP, Liu X, McKim DB, DiSabato DJ, Oliver B (2022). Dynamic interleukin-1 receptor type 1 signaling mediates microglia-vasculature interactions following repeated systemic LPS. J Inflamm Res.

[44] Redondo-Castro E, Cunningham C, Miller J, Martuscelli L, Aoulad-Ali S (2017). Interleukin-1 primes human mesenchymal stem cells towards an anti-inflammatory and pro-trophic phenotype in vitro. Stem Cell Res Ther.

[45] Guo DH, Yamamoto M, Hernandez CM, Khodadadi H, Baban B (2020). Visceral adipose NLRP3 impairs cognition in obesity via IL-1R1 on CX3CR1+ cells. J Clin Invest.

[46] Davalos D, Grutzendler J, Yang G, Kim JV, Zuo Y (2005). ATP mediates rapid microglial response to local brain injury in vivo. Nat Neurosci.

[47] Fan Y, Xie L, Chung CY (2017). Signaling pathways controlling microglia chemotaxis. Mol Cells.

[48] Fields RD (2011). Nonsynaptic and nonvesicular ATP release from neurons and relevance to neuron-glia signaling. Semin Cell Dev Biol.

[49] Butt AM (2011). ATP: a ubiquitous gliotransmitter integrating neuron-glial networks. Semin Cell Dev Biol.

[50] Krukowski K, Nolan A, Becker M, Picard K, Vernoux N (2021). Novel microglia-mediated mechanisms underlying synaptic loss and cognitive impairment after traumatic brain injury. Brain Behav Immun.

[51] Wogram E, Wendt S, Matyash M, Pivneva T, Draguhn A (2016). Satellite microglia show spontaneous electrical activity that is uncorrelated with activity of the attached neuron. Eur J Neurosci.

[52] Guo JZ, Graves AR, Guo WW, Zheng J, Lee A (2015). Cortex commands the performance of skilled movement. Elife.

[53] Otchy TM, Wolff SB, Rhee JY, Pehlevan C, Kawai R (2015). Acute off-target effects of neural circuit manipulations. Nature.

[54] Dayan E, Cohen LG (2011). Neuroplasticity subserving motor skill learning. Neuron.

[55] Fu M, Yu X, Lu J, Zuo Y (2012). Repetitive motor learning induces coordinated formation of clustered dendritic spines in vivo. Nature.

[56] Chen SX, Kim AN, Peters AJ, Komiyama T (2015). Subtype-specific plasticity of inhibitory circuits in motor cortex during motor learning. Nat Neurosci.

[57] Stemkowski PL, Smith PA (2012). Long-term IL-1beta exposure causes subpopulation-dependent alterations in rat dorsal root ganglion neuron excitability. J Neurophysiol.

[58] Viviani B, Boraso M, Marchetti N, Marinovich M (2014). Perspectives on neuroinflammation and excitotoxicity: a neurotoxic conspiracy?. Neurotoxicology.

[59] Wang S, Cheng Q, Malik S, Yang J (2000). Interleukin-1beta inhibits gamma-aminobutyric acid type A (GABA(A)) receptor current in cultured hippocampal neurons. J Pharmacol Exp Ther.

[60] Iori V, Frigerio F, Vezzani A (2016). Modulation of neuronal excitability by immune mediators in epilepsy. Curr Opin Pharmacol.

[61] Webster KM, Sun M, Crack P, O'Brien TJ, Shultz SR (2017). Inflammation in epileptogenesis after traumatic brain injury. J Neuroinflammation.

[62] Rodgers KM, Hutchinson MR, Northcutt A, Maier SF, Watkins LR (2009). The cortical innate immune response increases local neuronal excitability leading to seizures. Brain.

[63] Rossi S, Furlan R, De Chiara V, Motta C, Studer V (2012). Interleukin-1beta causes synaptic hyperexcitability in multiple sclerosis. Ann Neurol.

[64] Loddick SA, Rothwell NJ (1996). Neuroprotective effects of human recombinant interleukin-1 receptor antagonist in focal cerebral ischaemia in the rat. J Cereb Blood Flow Metab.

[65] Kelly A, Vereker E, Nolan Y, Brady M, Barry C (2003). Activation of p38 plays a pivotal role in the inhibitory effect of lipopolysaccharide and interleukin-1 beta on long term potentiation in rat dentate gyrus. J Biol Chem.

[66] Li Q, Qi F, Yang J, Zhang L, Gu H (2015). Neonatal vaccination with bacillus Calmette–Guerin and hepatitis B vaccines modulates hippocampal synaptic plasticity in rats. J Neuroimmunol.

[67] Tang Y, Feng B, Wang Y, Sun H, You Y (2020). Structure-based discovery of CZL80, a caspase-1 inhibitor with therapeutic potential for febrile seizures and later enhanced epileptogenic susceptibility. Br J Pharmacol.

[68] Fogarty MJ, Hammond LA, Kanjhan R, Bellingham MC, Noakes PG (2013). A method for the three-dimensional reconstruction of neurobiotin-filled neurons and the location of their synaptic inputs. Front Neural Circuits.

[69] Thévenaz P, Ruttimann UE, Unser M (1998). A pyramid approach to subpixel registration based on intensity. IEEE transactions on image processing: a publication of the IEEE Signal Processing Society.

[70] Zhang J, Zhang Q, Lou Y, Fu Q, Chen Q (2018). Hypoxia-inducible factor-1alpha/interleukin-1beta signaling enhances hepatoma epithelial-mesenchymal transition through macrophages in a hypoxic-inflammatory microenvironment. Hepatology.

[71] Giordano N, Iemolo A, Mancini M, Cacace F, De Risi M (2018). Motor learning and metaplasticity in striatal neurons: relevance for Parkinson’s disease. Brain.

[72] Rustay NR, Wahlsten D, Crabbe JC (2003). Influence of task parameters on rotarod performance and sensitivity to ethanol in mice. Behav Brain Res.

[73] Nakamura K, Moorhouse AJ, Cheung DL, Eto K, Takeda I (2019). Overexpression of neuronal K(+)-Cl(−) co-transporter enhances dendritic spine plasticity and motor learning. J Physiol Sci.

[74] Zheng Y, Zhang X, Wu X, Jiang L, Ahsan A (2019). Somatic autophagy of axonal mitochondria in ischemic neurons. J Cell Biol.

[75] Yu Z, Guindani M, Grieco SF, Chen L, Holmes TC (2022). Beyond *t* test and ANOVA: applications of mixed-effects models for more rigorous statistical analysis in neuroscience research. Neuron.

[76] Lazic SE, Clarke-Williams CJ, Munafo MR (2018). What exactly is ‘N’ in cell culture and animal experiments?. PLoS Biol.

