# Supplementary material for: Cell-specific IL-1R1 regulates the regional heterogeneity of microglial displacement of GABAergic synapses and motor learning ability
Source: Cell Mol Life Sci. 2024 Mar 4;81(1):116. doi: 10.1007/s00018-023-05111-0 (PMC10912170; doi:10.1007/s00018-023-05111-0)
Supplement: Supplementary file 7 — (DOCX 98922 KB) [file 18_2023_5111_MOESM7_ESM.docx]

***Submitted to Cellular and Molecular Life Sciences***

**Cell-specific IL-1R1 regulates the regional heterogeneity of microglial displacement of GABAergic synapses and motor learning ability**

YOU Yi^#1^, AN Da-dao^#1^, WAN Yu-shan^1^, ZHENG Bai-xiu^1^, DAI Hai-bin^1^, ZHANG She-hong^3^, ZHANG Xiang-nan^1^, Wang Rong-rong^4^, SHI Peng^1^, JIN Mingjuan^5^, WANG Yi^1,2^, JIANG Lei^1, *^, CHEN Zhong^1,2^, HU Wei-wei^1, *^

^1^Department of Pharmacology and Department of Pharmacy of the Second Affiliated Hospital, Key Laboratory of Medical Neurobiology of The Ministry of Health of China, School of Basic Medical Sciences, Zhejiang University School of Medicine, Hangzhou, 310058, China.

*^2^*Key Laboratory of Neuropharmacology and Translational Medicine of Zhejiang Province, Zhejiang Chinese Medical University, Hangzhou, 310053, China

^3^Department of Rehabilitation Medicine, Huzhou Central Hospital, Affiliated Huzhou Hospital, Zhejiang University School of Medicine, Huzhou, 313000, China

^4^Department of Clinical Pharmacy, The First Affiliated Hospital, Zhejiang University School of Medicine, Hangzhou

^5^Department of Epidemiology and Biostatistics, Zhejiang University School of Public Health, Hangzhou, 310058, China.

*Correspondence to:

Wei-Wei Hu, Ph.D., Professor, Tel: +86-571-88208768; Fax: +86-571-88208228; email address: [huww@zju.edu.cn](mailto:huww@zju.edu.cn)

Lei Jiang, Ph.D., Associate Professor: +86-571-88208226; Fax: +86-571-88208226; email address: jiang_lei@zju.edu.cn

^#^ The authors contribute equally to this work.

**Supplementary figures and legends**

**
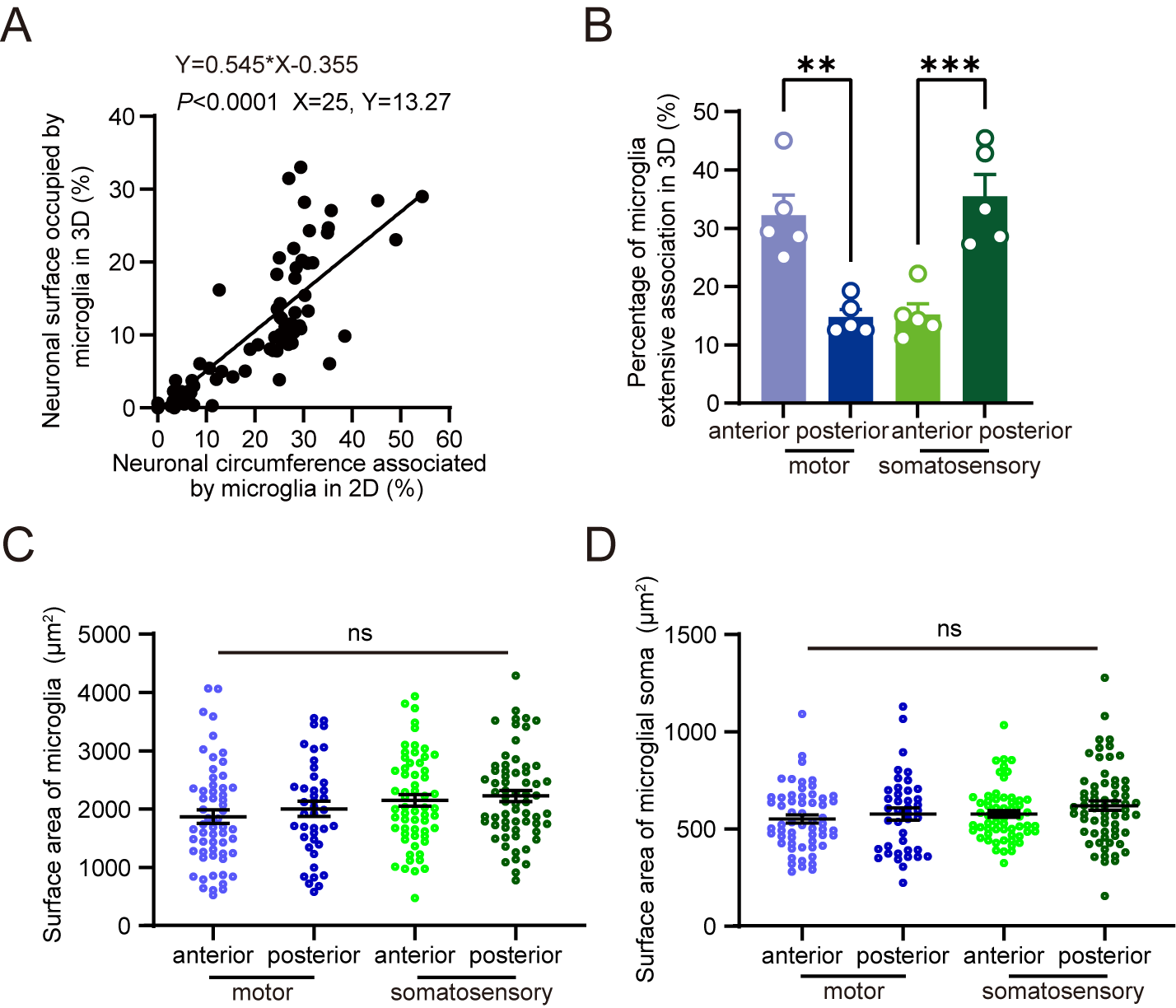
Supplementary Figure 1. The contact between microglia and neuronal soma in the motor cortex and somatosensory cortex in 3D reconstruction**. (A) Correlation analysis of the percentage of contact area for neurons in 3D reconstruction images and the percentage of neuronal circumference associated with microglia in 2D images. n=119 cells from 5 mice. (B) Quantification of the percentage of microglia extensively associated with neuronal soma in 3D reconstruction images. n=5 mice for each group. (C) The surface area per microglia across motor and somatosensory cortex. n=41-64 cells from 4 mice. (D) The surface area per microglial soma across motor and somatosensory cortex. n=41-64 cells from 4 mice. ***P* <0.01, ****P* <0.001.

**
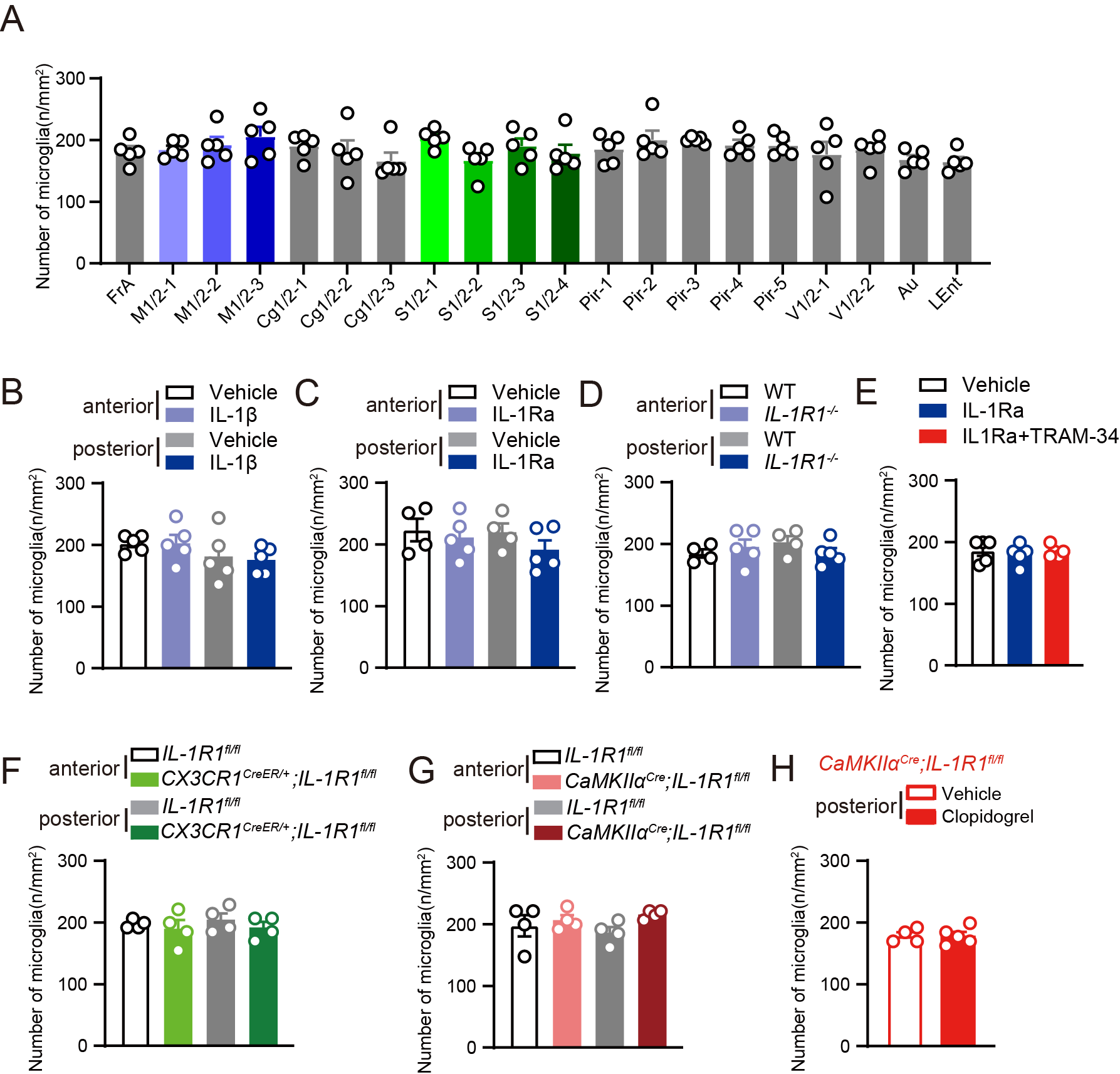
Supplementary Figure 2. The number of microglia in different groups.** (A) Number of microglia in different cortical brain regions. (B) Number of microglia in the IL-1β group and the vehicle group in WT mice. (C) Number of microglia in the IL-1Ra group and the vehicle group in WT mice. (D) Number of microglia in WT mice and *IL-1R1-/-* mice. (E) Number of microglia in WT mice given IL-1Ra or IL-1Ra combined with TRAM-34 in Mp. (F) Number of microglia in control mice (*IL-1R1^fl/fl^*) and *CX3CR1^CreER/+^;IL-1R1^fl/fl^* mice. (G) Number of microglia in control mice (*IL-1R1^fl/fl^*) and *CaMKIIα^Cre^;IL-1R1^fl/fl^* mice. (H) Number of microglia in vehicle or clopidogrel treated *CaMKIIα^Cre^;IL-1R1^fl/fl^* mice.

**
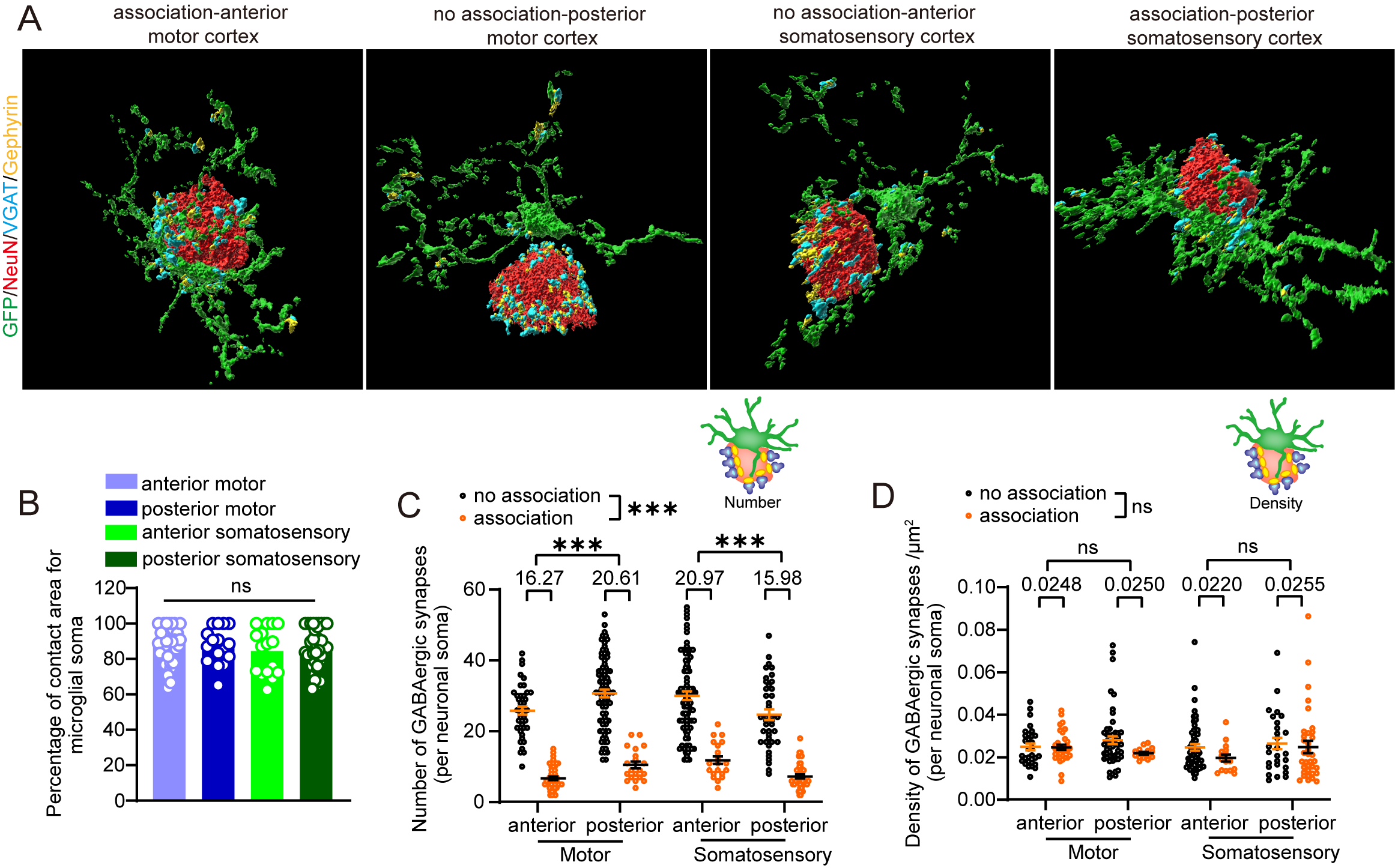
Supplementary Figure 3.** **The number and density of GABAergic synapses around neuronal soma and microglia in the motor cortex and somatosensory cortex in 3D reconstruction.** (A) 3D reconstruction images of GFP+ microglia (green), NeuN+ neurons (red), gephyrin+ (yellow) and VGAT+ (cyan) GABAergic synapses in motor cortex and somatosensory cortex. (B) The percentage of contact area from microglial soma for extensively association. n=16-35 cells from 3 mice. (C) The number of GABAergic synapses per neuronal soma which were closest to microglia. The average data are shown above the group. n=85-107 cells from 4 mice. (D) The density of GABAergic synapses on neuronal soma outside of contact area. The average data are shown above the group. n=62-70 cells from 3 mice. Association: extensively association between microglia and neurons; no association: no extensively association between microglia and neurons. ****P* <0.001.

**
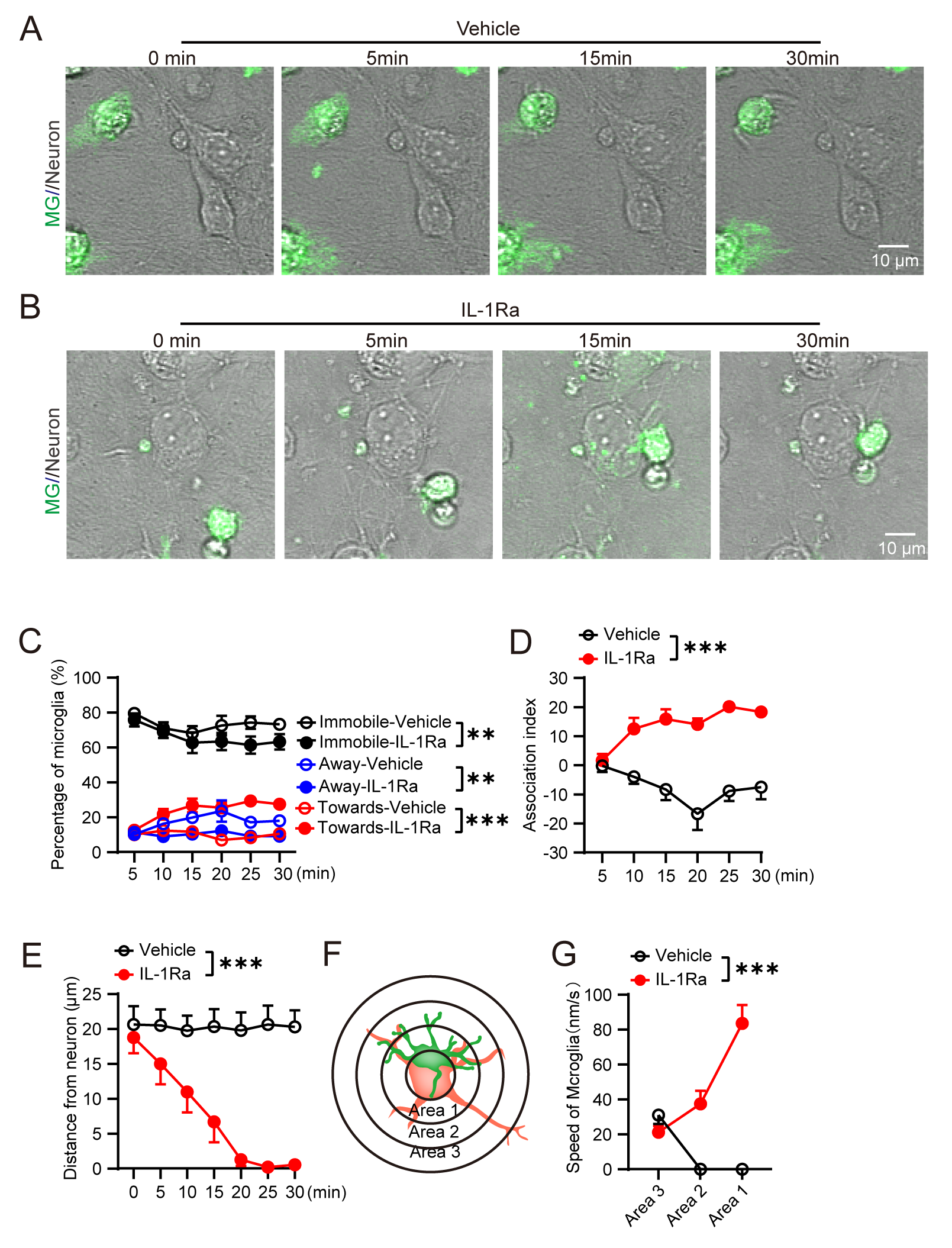
Supplementary Figure 4. Co-culture experiments of primary neurons and microglia.** (A-B) Confocal images of co-cultured primary neurons (grey) and GFP+ microglia (green) at a different time point after administration of vehicle (A) or IL-1Ra (B). (C) Percentage of microglia in different motion states: keeping immobile (Immobile), moving away from neurons (Away), or moving towards neurons (Towards) after administration of vehicle or IL-1Ra. (D) Association index of microglia, while moving towards neurons (1), moving away from neurons (-1), or immobile (0), after administration of vehicle or IL-1Ra. (E) Distance between microglia and neurons after administration of vehicle or IL-1Ra. (F) Schematic diagram of speed analysis. Microglial speed was calculated at 3 positions: one (Area 1), two (Area 2), and three (Area 3) neuronal diameters away from the neuron soma. (G) Moving speed of microglia towards neuron after administration of IL-1Ra or vehicle. n=9 neurons from 3 replicates. ***P* <0.01, ****P* <0.001.

**
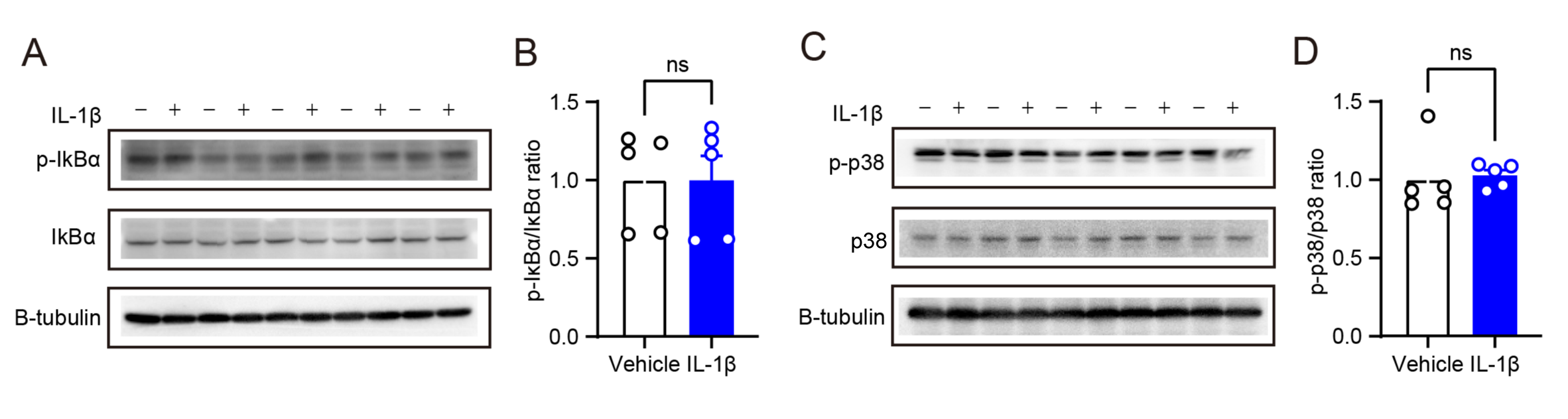
Supplementary Figure 5. Effects of IL-1β on phosphorylation of IκBα and p38 in anterior motor cortex.**  (A) Immunoblotting imaging of IκBα and p- IκBα. (B) Quantification of p- IκBα/ IκBα for the assessment of the inflammatory activation. (C) Immunoblotting imaging of p38 and p-p38. (D) Quantification of p-p38/p38 for the assessment of inflammatory activation. n=5 mice in each group.

**
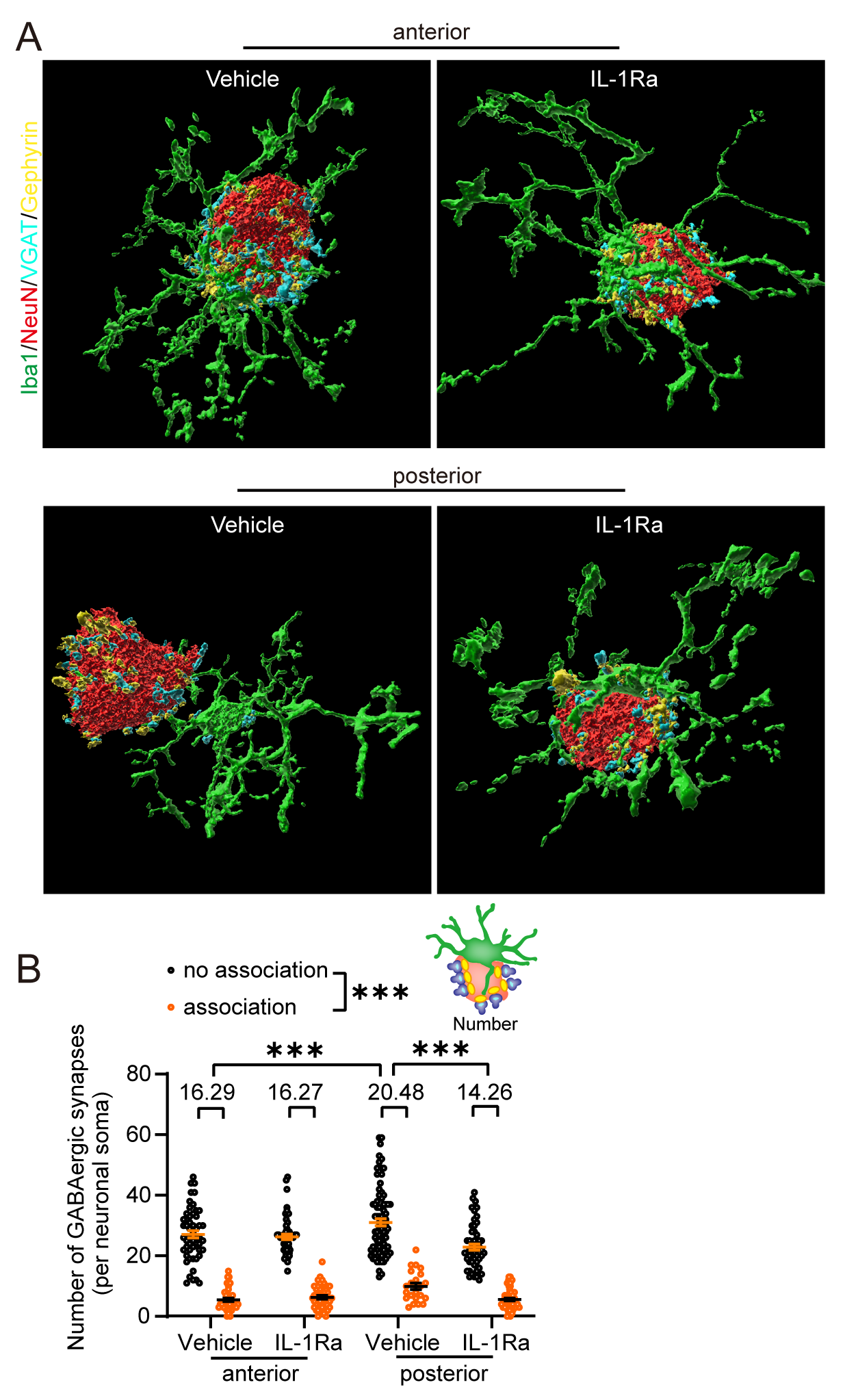
**

**Supplementary Figure 6. The number of GABAergic synapses on neuronal soma in 3D reconstruction after administration of IL-1Ra.** (A) 3D reconstruction images of Iba1+ microglia (green), NeuN+ neurons (red), gephyrin+ (yellow) and VGAT+ (cyan) GABAergic synapses in the anterior motor cortex (anterior) and posterior motor cortex (posterior) of IL-1Ra or vehicle-treated WT mice. (B) The number of GABAergic synapses per neuronal soma which were closest to microglia of IL-1Ra or vehicle-treated WT mice. The average data are shown above the group. n=95-107 cells from 4 mice in each group. ****P* <0.001.

**
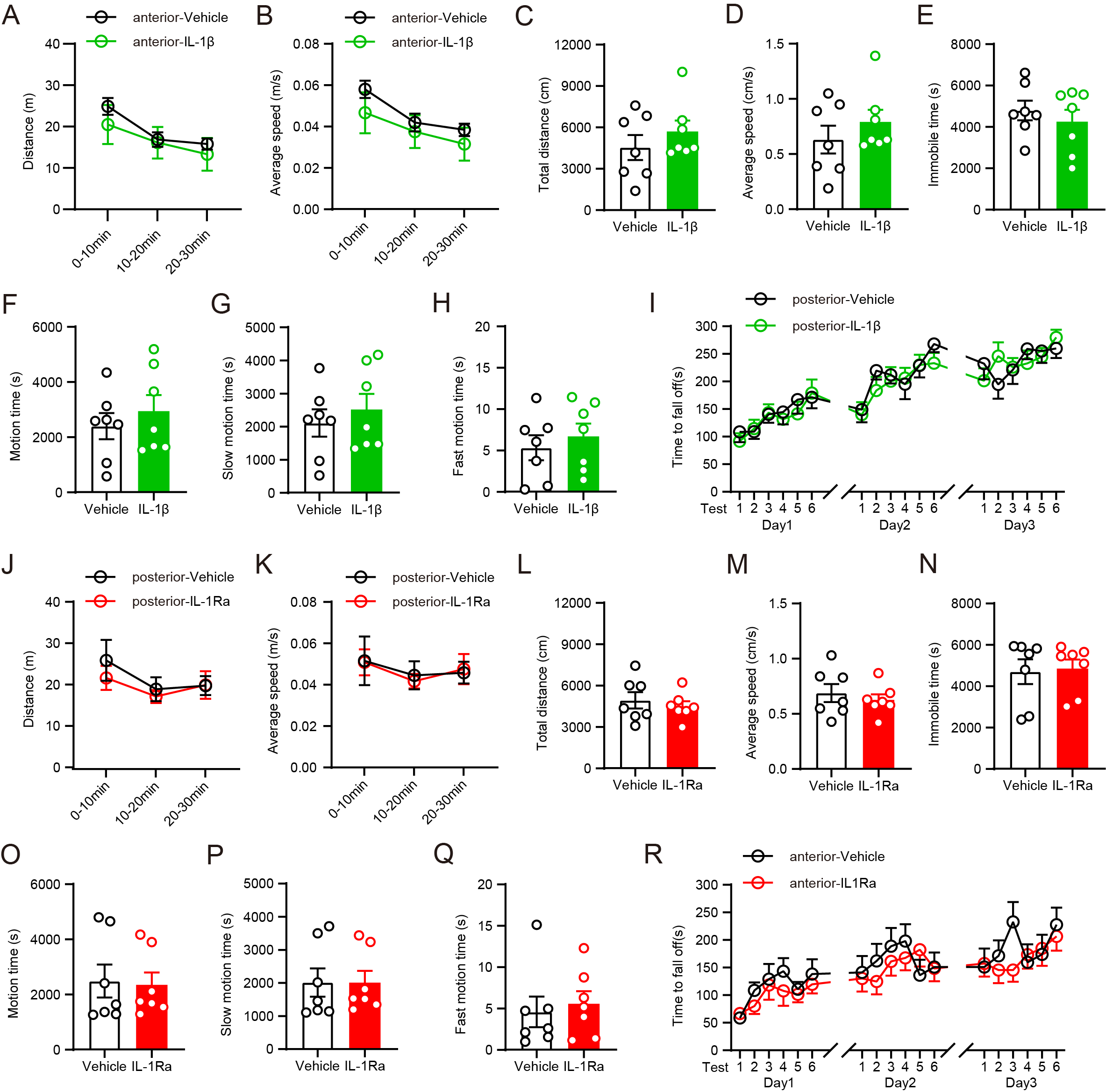
Supplementary Figure 7. IL-1β/IL-1R1 do not affect locomotor activity.** (A-B) Locomotor activity in an open field after administration of vehicle or IL-1β in the anterior motor cortex. The total distance (A) and average speed (B) traveled in the open field for every 10 min were quantified. n=7 mice. (C-H) Locomotor activity in the home cage after administration of vehicle or IL-1β in the anterior motor cortex. n=7 mice. (C) Total distance traveled in the home cage. (D) Average speed traveled in the home cage. (E) Immobile time when mice remained in the home cage. (F) Motion time when mice remained in the home cage. (G) Motion time when mice moved slowly in the home cage. (H) Motion time when mice moved fast in the home cage. (I) The latency to fall off in rotarod tests after administration of vehicle or IL-1β in the posterior motor cortex. n=8 mice. (J-K) Locomotor activity in an open field after administration of vehicle or IL-1Ra in the posterior motor cortex. Total distance (J) and average speed (K) traveled in the open field every 10 min were quantified. n=7 mice. (L-Q) Locomotor activity in the home cage after administration of vehicle or IL-1Ra in the posterior motor cortex. n=7 mice. (L) Total distance traveled in the home cage. (M) Average speed traveled in the home cage. (N) Immobile time when mice remained in the home cage. (O) Motion time when mice remained in the home cage. (P) Motion time when mice moved slowly in the home cage. (Q) Motion time when mice moved fast in the home cage. (R) The latency curve to fall off in rotarod tests after administration of vehicle or IL-1Ra in anterior motor cortex. n=8-9 mice.

**
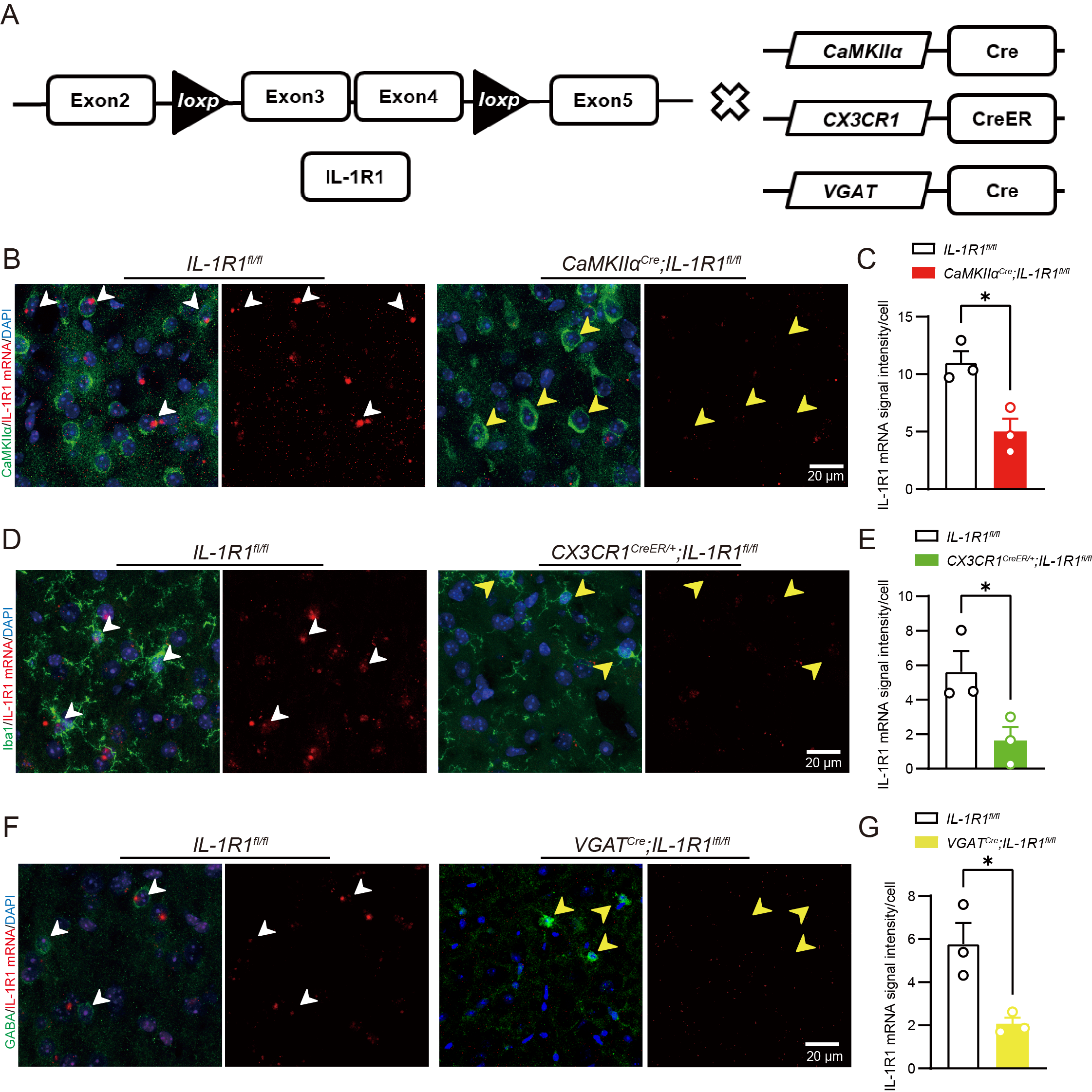
**

**Supplementary Figure 8. Specific deficit of IL-1R1 in different cell types by Cre-LoxP system**. (A) Schematic graph for the construction of *CaMKIIα^Cre^;IL-1R^lfl/fl^*, *CX3CR1^CreER/+^;IL-1R^lfl/fl^*, *VGAT^Cre^; IL-1R^lfl/fl^* mice. (B, D, F) Representative images of specific deficit of IL-1R1 in glutamatergic neurons (B), microglia (D), and GABAergic neurons (F). White arrowheads indicate cells with normal expression of IL-1R1 mRNA, yellow arrowheads indicate cells with deficient expression of IL-1R1 mRNA. (C, E, G) Quantification of the expression of IL-1R1 in glutamatergic neurons (C), microglia (E), and GABAergic neurons (G). n=3 mice in each group. **P*<0.05.

**
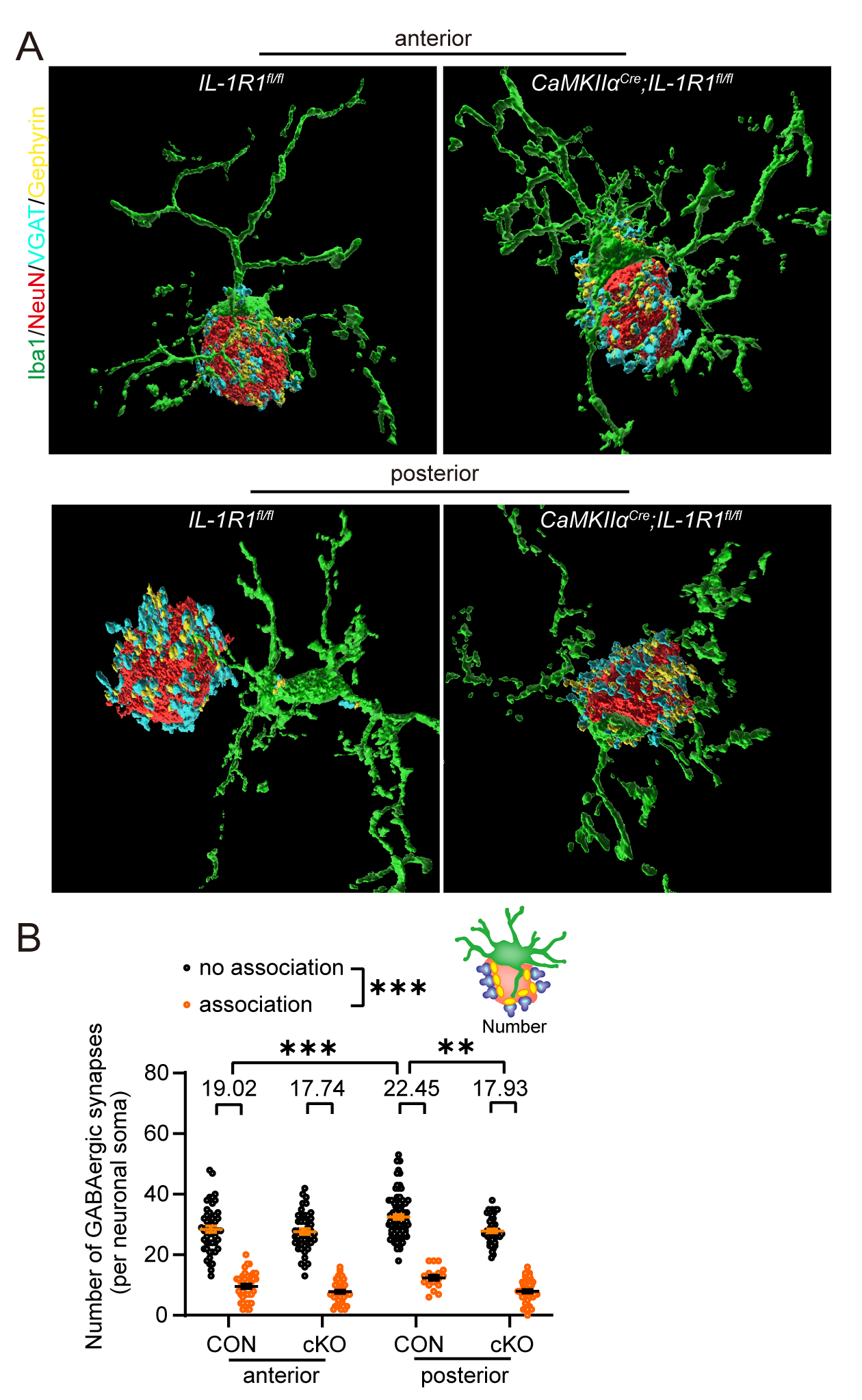
**

**Supplementary Figure 9. The number of GABAergic synapses around neuronal soma in 3D reconstruction after selective deficit of IL-1R1 in glutamatergic neurons.** (A) 3D reconstruction images of Iba1+ microglia (green), NeuN+ neurons (red), gephyrin+ (yellow) and VGAT+ (cyan) GABAergic synapses in the anterior motor cortex (anterior) and posterior motor cortex (posterior) of control mice *(IL-1R1^fl/fl^*) and *CaMKIIα^Cre^;IL-1R1^fl/fl^* mice. (B) The number of GABAergic synapses per neuronal soma which were closest to microglia of control mice *(IL-1R1^fl/fl^*, CON) and *CaMKIIα^Cre^;IL-1R1^fl/fl^* mice (cKO). n=89-97 cells from 4 mice in each group. ***P* <0.01, ****P* <0.001.

**
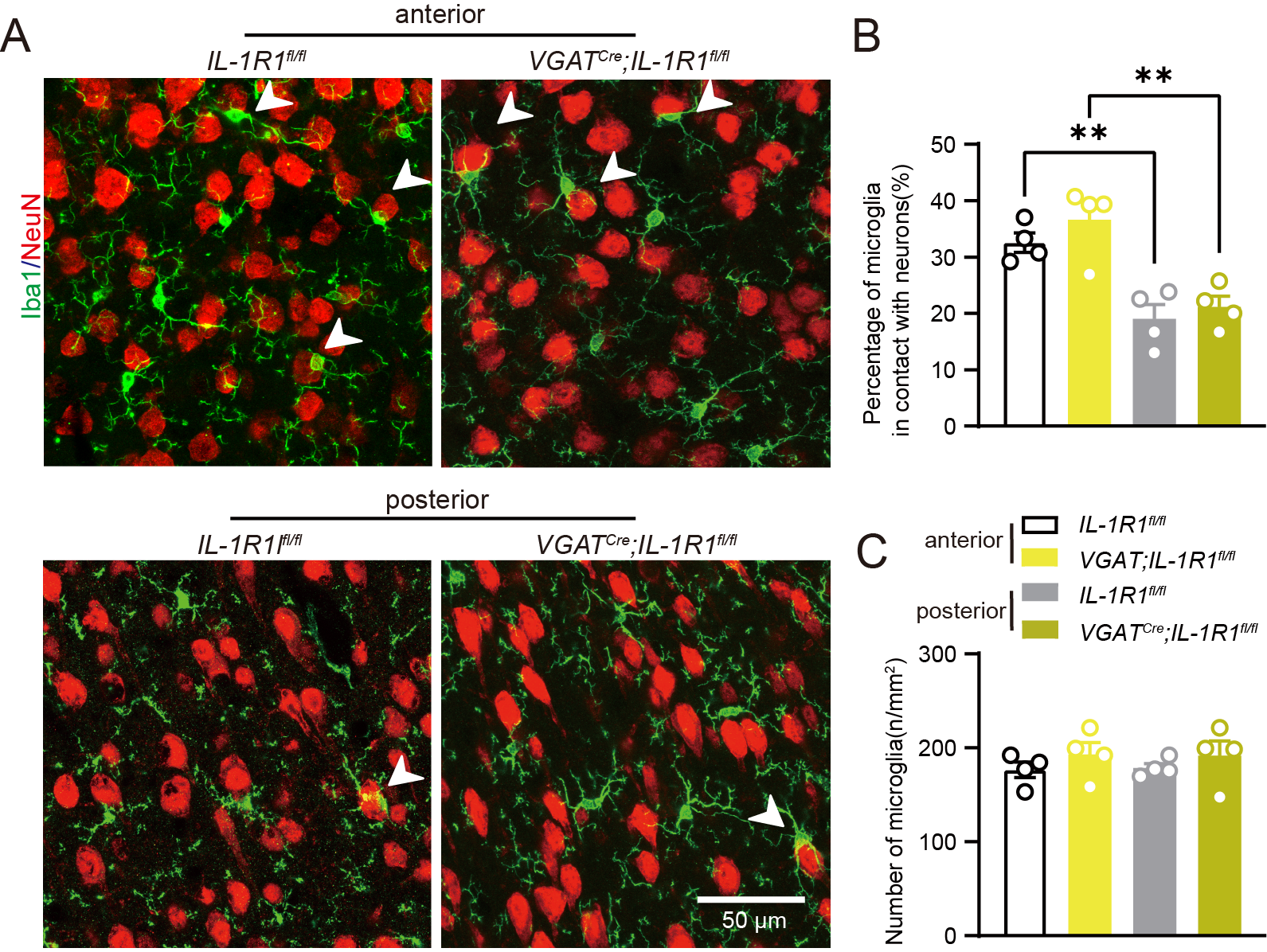
Supplementary Figure 10. Specific deficit of IL-1R1 in GABAergic neurons does not affect microglial association.** (A) Confocal images of Iba1+ microglia and NeuN+ neurons in control mice (*IL-1R1^fl/fl^*) and *VGAT^Cre^;IL-1R1^fl/fl^* mice. White arrowheads indicate microglia extensively associated with neuronal soma. (B) Percentage of microglia extensively associated with neurons in *IL-1R1^fl/fl^* and *VGAT^Cre^;IL-1R1^fl/fl^* mice. (C) Number of microglia in *IL-1R1^fl/fl^* and *VGAT^Cre^;IL-1R1^fl/fl^* mice. n=4 mice in each group. ***P* <0.01.

**Supplementary Video 1.** 3D reconstructions of GFP+ microglia (green) and NeuN+ neurons (red) in *CX3CR1^GFP/+^* mice. Microglia extensively associated with neurons in anterior motor cortex and posterior somatosensory cortex, but not extensively associated with neurons in posterior motor cortex and anterior somatosensory cortex. Related to Figure 1D.

**Supplementary Video 2.** 3D reconstructions of GFP+ microglia (green), VGAT+ GABAergic synapses (white), and NeuN+ neurons (red) in *CX3CR1^GFP/+^* mice. Microglia extensively associated with neurons in anterior motor cortex and posterior somatosensory cortex, but not extensively associated with neurons in posterior motor cortex and anterior somatosensory cortex. Related to Figure 1F.

**Supplementary Video 3.** Time lapse video of co-cultured primary neurons (with a white asterisk) and GFP+ microglia after administration of vehicle. 20x acceleration.

**Supplementary Video 4.** Time lapse video of co-cultured primary neurons (with a white asterisk) and GFP+ microglia after administration of IL-1Ra. 20x acceleration.

**Supplementary Video 5.** Time lapse video of GCAMP6(s) labeled glutamatergic neurons (green) either associated with microglia (red) or not for Figure 3E. The left neuron: extensively associated with microglia; the right neuron: not extensively associated with microglia. The frequency of calcium transients of glutamatergic neuron (left) extensively associated with microglia was higher than neuron (right) not extensively associated with microglia, but the intensity was comparable. 20x acceleration.

**Supplementary Video 6.** Time lapse video of GCAMP6(s) labeled glutamatergic neurons (green) during extensive association with microglia after administration of IL-1Ra for Figure 3I. Base: before administration of IL-1Ra; Pre: after IL-1Ra administration, but before extensive association with microglia; Extensive association: during extensive association with microglia; Post: the extensive association was attenuated. After administration of IL-1Ra, the frequency of calcium transients of neuron did not change before extensively associated with microglia (Pre), but became higher after extensively associated with microglia (Association). When the extensive association was attenuated (Post), the frequency of neuron returned to baseline (Base). The intensity did not change with time. 100x acceleration.
